# No role for initial severity on the efficacy of antidepressants: results of a multi-meta-analysis

**DOI:** 10.1186/1744-859X-12-26

**Published:** 2013-08-13

**Authors:** Konstantinos N Fountoulakis, Areti Angeliki Veroniki, Melina Siamouli, Hans-Jürgen Möller

**Affiliations:** 1 3rd Department of Psychiatry, School of Medicine, Aristotle University of Thessaloniki, 6, Odysseos str (1st Parodos Ampelonon str.), Pylaia, Thessaloniki 55535, Greece; 2 Department of Hygiene and Epidemiology, School of Medicine, University of Ioannina, Ioannina 45110, Greece; 3 Department of Psychiatry, Ludwig-Maximilians-Universität München, Nussbaumstrasse 7, Munich 80336, Germany

**Keywords:** Antidepressants, Depression, Meta-analysis, Effect size, Baseline severity

## Abstract

**Introduction:**

During the last decade, a number of meta-analyses questioned the clinically relevant efficacy of antidepressants. Part of the debate concerned the method used in each of these meta-analyses as well as the quality of the data set.

**Materials and methods:**

The Kirsch data set was analysed with a number of different methods, and eight key questions were tackled. We fit random effects models in both Bayesian and frequentist statistical frameworks using raw mean difference and standardised mean difference scales. We also compare between-study heterogeneity estimates and produce treatment rank probabilities for all antidepressants. The role of the initial severity is further examined using meta-regression methods.

**Results:**

The results suggest that antidepressants have a standardised effect size equal to 0.34 which is lower but comparable to the effect of antipsychotics in schizophrenia and acute mania. The raw HDRS difference from placebo is 2.82 with the value of 3 included in the confidence interval (2.21–3.44). No role of initial severity was found after partially controlling for the effect of structural (mathematical) coupling. Although data are not definite, even after controlling for baseline severity, there is a strong possibility that venlafaxine is superior to fluoxetine, with the other two agents positioned in the middle. The decrease in the difference between the agent and placebo in more recent studies in comparison to older ones is attributed to baseline severity alone.

**Discussion:**

The results reported here conclude the debate on the efficacy of antidepressants and suggest that antidepressants are clearly superior to placebo. They also suggest that baseline severity cannot be utilized to dictate whether the treatment should include medication or not. Suggestions like this, proposed by guidelines or institutions (e.g. the NICE), should be considered mistaken.

## Introduction

Recently, a number of meta-analytic studies questioned the clinical usefulness of antidepressants. It has been shown that there is a significant bias in the publication of antidepressant trials [1] and that the effect size of the medication group in comparison to that of the placebo is rather small [2-9]. On the basis of these results, a ‘conspiracy theory’ involving the Food and Drug Administration (FDA) was proposed [10,11]. Furthermore, by ‘overstretching’ the interpretation of the data, it has been suggested that because they do not incur drug risks, alternative therapies (e.g. exercise and psychotherapy) may be a better treatment choice for depression [10]. These triggered much interest from the mass media and from intellects outside the mental health area, often with a biased and ideologically loaded approach [12]. However, the most important suggestion was that initial severity plays a major role and antidepressants might not have any effect at all in mildly depressed patients [5,6,8].

Following this conclusion, several authors and agencies like the National Institute of Clinical Excellence (NICE) suggested the utilisation of ‘alternative’ treatment options (e.g. exercise and psychotherapy) in mildly depressed patients and pharmacotherapy only for the most severe cases. Among other things, these authors and authorities did not take into consideration that, peculiarly, similar findings were reported concerning psychotherapy [13-16].

Several authors criticised the above by focusing on the limitations of randomised clinical trials (RCTs), on clinical issues and, especially, on the problematic properties of the Hamilton depression rating scale (HDRS) and on the fact that the effectiveness of antidepressants in clinical practice is normally optimised by sequential and combined therapy approaches. It has been proposed that the effect is significant in a subgroup of patients [17]. So far, only two efforts were made to re-analyse the same data set with different methodological approaches [18,19]. These two efforts independently reported the results that are quite similar between them but different from those of the study of Kirsch et al.

All the meta-analytic studies mentioned above were based on five ‘data sets’. The data sets are the Khan et al. set [8,20], the Turner et al. set [1], the Kirsch et al. set [5], the Fournier et al. set [6] and the Undurraga and Baldessarini set [9].

All the meta-analyses are shown in Table 1 with respect to the methodology used and results. In this table, Undurraga and Baldessarini [9] was not included because these authors utilised a different outcome measure. The Fournier et al. [6] analysis was also not included because this data set is highly heterogeneous and includes primary care patients with dysthymia and major depressive patients who accepted to be randomised to medication, psychotherapy or placebo, fixed as well as flexible dosage studies and medication up to 50 mg of paroxetine but only up to 100 mg of imipramine [21-25]. It is interesting that a common denominator of the studies included in this specific meta-analysis was that the efficacy of psychosocial interventions depends also on initial severity, the same way the medication does. In the Unduraga and Baldessarini set, variance measures are missing in many trials. However, in the Khan et al. data set, only 21 out of 45 studies reported a standard error of measurement or a standard deviation of mean change. The data of the Turner et al. set are not available to the authors of the current paper except for the effect sizes of individual studies. On the other hand, the Kirsch et al. set is more complete and available online.

**Table 1:** Estimation of the overall effectiveness and magnitude of heterogeneity

**Analyses**	**Effect size (CI/CrI)**		**Heterogeneity (CrI)**		**Role for initial severity**		**Respects randomisation**
	**RMD**	**SMD**	**RMD**	**SMD**	**RMD**	**SMD**	
Frequentist methods							
Simple random effects meta-analysis	2.71 (1.96, 3.45)	0.33 (0.24, 0.42)	2.50	0.03			Yes
NMA random effects analysis			2.31	0.03			Yes
Meta-regression random effects analysis			1.57	0.02	Yes	Yes	Yes
Turner et al. (random effects meta-analysis)	-	0.31 (0.27, 0.35)	-	-	-	-	Yes
Khan et al., ratio of the number of early discontinued patients divided by the total number of patients in each group (chi-square is also calculated)	-	-	-	-	Yes	Yes	No
Kirsch et al. (weight by sample size/SD^2^)	1.80	0.32	-	-	-	Yes	No
Horder et al. (random effects meta-analysis)	2.70 (1.95, 3.44)	-	-	-	Yes	-	Meta-analysis: yes
							Meta-regression: no
Fountoulakis et al. (sample size weighting)	2.18	0.32	-	-	-	-	No
Fountoulakis et al. (inverse variance weighting)	2.68	0.35	-	-	-	-	No
Bayesian methods							
Simple random effects meta-analysis	2.61 (1.94, 3.30)	0.32 (0.25, 0.40)	1.61 (0.53, 3.53)	0.02 (0.00, 0.05)			Yes
NMA random effects analysis			0.71 (0.24, 0.98)	0.01 (0.00, 0.05)			Yes
Simple random effects meta-regression analysis	2.77 (2.18, 3.36)	0.34 (0.27, 0.42)	0.58 (0.00, 2.13)	0.01 (0.00, 0.05)	Yes	No	Yes
Simple random effects meta-regression analysis using 12 different priors					Yes	No	Yes
NMA random effects meta-regression analysis			0.59 (0.01, 2.19)	0.01 (0.00, 0.05)	Yes	No	Yes
NMA random effects meta-regression using 12 different priors					Yes	No	Yes

Using the raw mean difference (RMD) and standardised mean difference (SMD) under both frequentist and Bayesian methods. In the frequentist approach, each variable is provided with its confidence interval (CI) and in the Bayesian methods with its credible interval (CrI). Each method is categorised according to its association with the role for initial severity and its respect to randomisation.

The data set of Kirsch et al. [5] might serve as a paradigm since it has been independently re-analysed by two other groups [18,19] and is based on FDA data which seem to be free of bias [26]. Thus, the current study will utilise the Kirsch et al. (reference) data set and will focus on the debate following its analysis and re-analysis.

It is important to define the specific questions that arise from the debate. According to our judgement, they are the following:

1. What is the bias in the Kirsch data set? How complete is this data set?

2. What is the magnitude of the heterogeneity (*τ*^2^) of the studies in this data set?

3. Which is the most appropriate method for meta-analysis of this data set?

4. What is the standardised mean difference (SMD) for the efficacy of antidepressants vs. placebo?

5. What is the raw HDRS mean difference (RMD) for the efficacy of antidepressants vs. placebo?

6. Is the SMD or the raw score more appropriate to reflect the difference between the active drug and the placebo?

7. Are all antidepressants equal in terms of efficacy?

8. What is the role of the initial severity?

9. Is there a change in the difference between active drug and placebo in more recent RCTs in comparison to older ones?

There is some hierarchical interrelationship between the aforementioned questions, which requires sequential answers in order to clarify the issue. The current paper will tackle these questions and will try to provide answers with the use of multiple methods of meta-analysis.

## Materials and methods

The Kirsch et al. database as published by these authors [27] was used in the current analysis. The complete set used in the current study is shown in Additional file 1.

Since one element of the debate was the use of different methods of meta-analysis, a number of methods were used in the current study and their results were compared. These were (a) simple random effects (RE) meta-analysis (simple REMA), (b) network RE meta-analysis (NMA), (c) simple RE meta-regression and (d) NMA RE meta-regression, in both Bayesian and frequentist frameworks. The description, advantages and disadvantages of each of these methods can be found in Additional file 2.

All approaches have been undertaken under the RE model [28-30], so as to account for between-study heterogeneity due to the differences in the true effect sizes, rather than chance. We selected the RE meta-analysis since our prior belief was that treatment effects vary across studies, and our aim was to infer on the distribution of the effects. In case there is no statistical variability in the effects, RE model simplifies to fixed effects model with *τ*^2^ equal to zero. We further applied meta-regression methods for the synthesis of the data, as it allows for the inclusion of study-level covariates that may explain the presence of heterogeneity. We explored whether two moderators, the initial severity and publication year, were associated with the treatment effect. One of the studies in the database was considered by Kirsch et al. to be an outlier. We therefore performed all meta-regression analyses with and without this particular study. In NMA models, we ranked all antidepressants using the probability of being the best [31] in the frequentist setting and the cumulative ranking probabilities in the Bayesian framework [32]. All methods were carried out employing both RMD and SMD scales.

The main differences between Bayesian and frequentist methods regard the estimation of heterogeneity. In meta-analysis, the choice of the method for estimating heterogeneity is a great issue since imprecise or biased approaches might lead to invalid results. Several methods have been suggested for estimating heterogeneity. In the frequentist methods, we estimated a ‘fixed’ parameter of the heterogeneity and we employed the commonly used DerSimonian and Laird (DL), or in case DL was not available, we performed the popular restricted maximum likelihood estimator. In the Bayesian framework, we accounted for the uncertainty in the estimation of heterogeneity, assuming it is a random variable. The magnitude of uncertainty associated with heterogeneity is included in the results and may have a considerable impact on our inferences. However, the Bayesian estimation of the heterogeneity under different prior selections for *τ*^2^ can be shown problematic when few studies are available [33,34]. We therefore consider 12 different prior distributions for the heterogeneity in the NMA RE meta-regression model so as to evaluate any possible differences in the results.

## Results

The complete results of the analyses are shown in Additional file 3.

### What is the bias in the Kirsch data set? How complete is this data set?

The funnel plots (Section 1 in Additional file 3) according to both RMD and SMD, treatment effects suggest that there is no asymmetry in the way the data points lie within the region defined by the two diagonal lines, which represent the 95% confidence limits around the summary treatment effect. Thus, there is no evidence for the presence of bias, as both funnel plots are visually symmetrical.

### What is the magnitude of the heterogeneity of the studies in this data set?

All RMD analyses showed the presence of important heterogeneity, and all RMD Bayesian approaches apart from simple RE meta-regression analysis showed that *τ*^2^ is significantly greater than zero. On the contrary, SMD exhibited lower and not statistically significant heterogeneity. This is in agreement with previous empirical findings [35] suggesting that SMD is more consistent than RMD as baseline varies. To investigate the presence of heterogeneity, we employed the RE meta-regression analysis with initial severity as a covariate. The RMD RE meta-regression analysis reduced the magnitude of heterogeneity, suggesting that initial severity explains part of the magnitude of the heterogeneity, whereas SMD suggests that initial severity does not play a significant role in the variance of the treatment effects.

The magnitude of heterogeneity when the SMD RE meta-regression model was employed with 12 different prior distributions for *τ*^2^ ranged in between 0.00 and 0.04 with all cases apart from the weakly informative gamma prior distribution being not statistically significant. However, the RMD heterogeneity ranged in between 0.24 and 1.29 with all credible intervals, apart from the two non-informative uniform priors for the logarithm of *τ*^2^, being significantly greater than zero. We therefore observe that RMD scale is sensitive in the prior selection of *τ*^2^, which impacts on the results and may lead to different statistical inferences. The two scales suggest different results regarding the magnitude of heterogeneity due to their different properties. The heterogeneity of the data according to different methods is shown in detail in Section 2 in Additional file 3. The estimation of heterogeneity is important in choosing the appropriate model for the analysis of data [33,34].

### Which is the most appropriate method for meta-analysis of this data set?

The selection of the effect size relying only on the magnitude of heterogeneity is not appropriate and can be shown problematic. It is suggested that the choice of the effect measure should be guided by empirical evidence and clinical expertise. Empirical investigations have shown that the SMD scale is less heterogeneous than RMD and that gives more reliable results as baseline risks vary, which is in agreement with our findings. However, it has been found that the SMD for small trials (number of included patients per group less than 10) bias the results towards to the null value in around 5%–6% of the cases even when the small sample correction factor is used [35]. Although this bias can contribute to the decreased heterogeneity of SMD, in our data set, all study arms apart from one included more than 10 patients. In our different analyses, the SMD scale was more consistent than the RMD, suggesting more valid results.

Although simple RE meta-analysis provides the most reliable evidence, it only gives insights on the effectiveness between the two treatments. Our data set includes evidence on multiple interventions, and the need to compare and rank these treatments suggests the use of NMA. However, the presence of heterogeneity in NMA analysis should be investigated. We therefore explore any possible reasons for its presence by employing NMA RE meta-regression with initial severity as covariate. However, since initial severity forms part of the definition of both SMD and RMD, there is a strong relationship between the covariate and the effect size (mathematical coupling). It is therefore very likely in the frequentist setting to find a significant relationship between initial severity and treatment effectiveness. In the Bayesian setting though, we ‘correct’ for this artefact by adjusting towards the global mean [36-39]. In the Bayesian NMA RE meta-regression model, we assume a fixed coefficient (*β*) for all treatment comparisons and we assign to it an uninformative prior. The method is more powerful than carrying out several independent pairwise meta-regressions.

We therefore conclude that Bayesian NMA RE meta-regression model using the most consistent scale (SMD) is the most appropriate method to meta-analyse these data.

### What is the SMD for the efficacy of antidepressants vs. placebo?

The SMD in simple RE meta-analysis under the frequentist approach is 0.33 (0.24–0.42) and under the Bayesian approach is 0.32 (0.25–0.40). Accounting for initial severity in all antidepressants, we apply a simple RE meta-regression analysis reflecting an SMD under the Bayesian approach at 0.34 (0.27–0.42), which does not change after the omission of the outlier study. In essence, all methods give a similar SMD value (see Sections 4 and 5 in Additional file 3).

### What is the raw HDRS mean difference for the efficacy of antidepressants vs. placebo?

The RMD in the simple RE meta-analysis under the frequentist approach is 2.71 (1.96–3.45) and under the Bayesian approach is 2.61 (1.94–3.30). We investigate the relationship between initial severity and treatment efficacy via the simple RE meta-regression analysis which under the Bayesian approach gives an RMD at 2.77 (2.18–3.36). After excluding the outlier, the raw HDRS value is 2.82 (2.21–3.44) (see Sections 4 and 5 in Additional file 3).

Again, all methods give a similar result, and all confidence intervals extend above the value of 3 which represents the NICE criterion for clinical relevance.

### Is the SMD or the raw score more appropriate to reflect the difference between the active drug and the placebo?

As written above, the use of SMD with a Bayesian approach would be the most appropriate method to meta-analyse these data, since it is associated with the least heterogeneity.

### Are all antidepressants equal in terms of efficacy?

The comparison of antidepressants with placebo as reference suggests that according to all methods used, all antidepressants are superior to placebo.

Venlafaxine is probably the most effective followed by paroxetine, while fluoxetine is the least effective according to all analyses, except for NMA RE meta-regression using RMD that suggests venlafaxine and nefazodone are similar and more effective than the others.

The hierarchical classification of agents has been done by the use of SUCRA values in the Bayesian analysis [40] or the posterior probabilities in the frequentist analysis [31]. Although both methods give insight on the ranking of treatments, the Bayesian approach using SUCRA values would be the most valid method. The main difference between SUCRA values and the probability of each treatment to be the best is that the former takes into account the uncertainty around the mean of the distribution of the effects, whereas the latter relies only on the mean of the distribution. Although confidence intervals overlap, SUCRA values give a strong probability of which agent performs better. The fact that confidence intervals overlap puts doubt on whether there is a true difference between agents.

NMA methods using RMD measure suggest that fluoxetine is clearly inferior to venlafaxine, since credible intervals of these two agents do not overlap. Similarly, the SMD scale suggests that venlafaxine is superior to all antidepressants but not significantly different (see Section 7 in Additional file 3).

### What is the role of the initial severity?

When the RMD is used in the calculations, both frequentist and Bayesian methods suggest a significant influence of the initial severity. This, also, explains the reduction of the amount of the heterogeneity from simple RE meta-analysis to simple RE meta-regression and from NMA to NMA RE meta-regression analysis.

However, when the SMD is considered, the frequentist simple RE meta-regression suggests a significant influence for initial severity, while in contrast, the Bayesian methods (simple RE meta-regression and NMA RE meta-regression) suggest no such an influence exists. It is possible that different effect sizes can lead to different inferences regarding baseline. Relying on the Cochrane Handbook, the effect size that is ‘close to no relationship with baseline risk is generally preferred for use in meta-analysis’. Moreover, the investigation of the relationship between treatment effects and initial severity under the frequentist methods can lead to inappropriate results, since they are inherently correlated [36]. However, the use of uninformative prior distribution for the regression coefficient and the adjustment for the mean baseline in the Bayesian setting relaxes the strong correlation between the treatment effect and the initial severity resulting in more reliable inferences for this relationship [36]. The results under the SMD effect measure suggest that there is no significant role of initial severity in the treatment outcome.

### Is there a change in the difference between active drug and placebo in more recent RCTs in comparison to older ones?

Although there seems to be a change in the difference between the active drug and placebo in more recent RCTs in comparison to older ones, the use of simple RE meta-regression with two covariates (initial severity and year of publication) using either RMD or SMD suggests that the year of publication is not important while initial severity is. This means that the attenuated difference can be attributed to a lower initial severity in newer RCTs in comparison to older ones.

The use of the ‘year of publication’ is an arbitrary variable. Alternatively, we could have used only the two last digits of it or the years since the oldest trial included. At any case, this analysis gives only a hint that initial severity is important and not the years passed (reflecting change in other factors). A method to quantify the years passed (except from the arbitrary year of publication) is an unanswered question.

## Discussion

For the last 10 years, after the Khan et al. meta-analysis, and especially after the Kirsch et al. publication [5,8], the efficacy of antidepressants in the treatment of major depression was under dispute. The current multi-meta-analysis utilised the Kirsch et al. data set and suggests that the most appropriate methods to meta-analyse these data are RE meta-regression models in a Bayesian setting using the SMD scale. It is important to decide which method of meta-analysis is best for the current data set, since different methods and different effect measures have different properties and can therefore result in different estimates [35,41,42].

The use of SMD in a Bayesian RE meta-regression model suggests that the standardised effect size of antidepressants relative to placebo is 0.34 (0.27–0.42), and there is no significant role for the initial severity of depression. The most probable raw HDRS change score is 2.82 (2.21–3.44) extending above 3. Our analysis showed that antidepressants are not equally effective. Bayesian NMA approaches suggest that venlafaxine is more effective than the rest with fluoxetine being the least effective among antidepressants.

The Kirsch hypothesis concerning depression is that there is a response which lies on a continuum from no intervention at all (e.g. waiting lists) to neutral placebo, then to active and augmented placebo including psychotherapy and finally to antidepressants which exert a slightly higher efficacy probably because blinding is imperfect because of the side effects (enhanced placebo) [10,43-48]. The full theory of Kirsch and its criticism can be found elsewhere [49,50].

The meta-analytical methods applied so far have advantages and limitations and much of the discussion focused on these limitations, and biases are introduced (Table 1). In the analysis of Kirsch et al. [5], the authors calculated the mean in drug change and the mean in placebo change and then took their difference. This breaks the randomisation and introduces bias, as it ignores the studies' characteristics and the sample size [51-53]. The so-called naïve comparisons are liable to bias and overprecise estimates. Horder et al. [19] used simple meta-analysis in a frequentist approach. They used standard meta-analytic approaches (fixed and random effects meta-analysis) and applied meta-regression in frequentist approach where the drug change vs. placebo change is plotted. Meta-regression, the way they used it, also breaks the randomisation as it does not account for the correlation between the change in placebo and the change in drug. Fountoulakis and Moller [18] used two methods: (a) sample size weighting which is appropriate when a set of independent effect sizes (e.g. RMD, SMD) is combined, but again, it breaks the randomisation and introduces bias. (b) Inverse variance weighting which applies weight as the inverse variance or the precision of each arm in each study. The precision of the effect estimates is the most accurate estimation of the summary effect size. It calculates the standardised change both for drug and placebo and then takes their difference. However, this again breaks the randomisation and introduces bias. Khan et al. [8] applied simple regression in frequentist approach where the drug change vs. baseline is plotted and the correlation coefficient is calculated. However, the precision of each study and the heterogeneity is not taken into account as in a meta-regression analysis. Then, in order to draw conclusions, the authors divided the sum of the number of early discontinued patients by the sum of the number of total patients in each arm and then calculated the chi-square. This is not an appropriate analysis as it also breaks the randomisation.

We believe that the current paper resolves the debate concerning the efficacy of antidepressants and its possible relationship to the initial severity in a definite manner.

The argument that an SMD of 0.30–0.35 is a weak one and suggests that the treatment is not really working or it does not make any clinically relevant difference neglects the fact that such an effect size is the rule rather than the exception [54]. Traditionally, an SMD of around 0.2 is considered to be small, around 0.5 is considered medium and around 0.8 is considered to be large [55], but this is an arbitrary assumption. However, in the real world of therapeutics, things are quite different. For comparison, one should look at the acute mania meta-analyses which suggest an SMD of 0.22 [56] or 0.42 [57], while clinically, acute mania is one of the easiest-to-treat acute psychiatric conditions. Also, the SMD of antipsychotics against the positive symptoms of schizophrenia is 0.48 [58].

The present study suggests that in this data set, the SMD results in more meaningful inferences than the RMD effect measure, since a greater amount of heterogeneity is produced using RMD. However, all calculations of RMD suggested a mean close to 3 and confidence intervals including the value of 3, thus suggesting that the RMD is not lower than the suggested NICE criterion. However, this criterion is arbitrary and unscientific, both in terms of clinical experience as well as in mathematical terms (because of the mathematical coupling phenomenon, see below), but this discussion is beyond the scope of the current paper [59,60].

Because the earlier meta-analyses suggested that initial severity is related to outcome with more severe cases responding better to antidepressants in comparison to placebo, some authors suggested that medication might not work at all for mildly depressed patients. Thus, they argued that for these patients, medication should not be prescribed; instead, alternative treatments which presumably lack side effects should be preferred, in spite of the possibility that the difference between medication and psychotherapy is similar to that between medication and placebo [61]. The suggestion to avoid pharmacotherapy in cases of mild depression is adopted also by the most recent NICE guidelines CG90. An immediate consequence of this is that patients suffering from mild depression are deprived from receiving antidepressants, on the basis of this conclusion and the overvaluation of ‘alternative therapies’.

‘Common sense’ among physicians leads to the belief that patients with greater disease severity at baseline respond better to treatment. The relation between baseline disease severity and treatment effect has a generic name in the statistical literature: ‘the relation between change and initial value’ [62] because treatment effect is evaluated by measuring the change of variables from their initial (baseline) values. In psychology, it is also well known as the ‘law of initial value’ [63].

However, the concept of ‘mathematical coupling’ , which was demonstrated for the first time by Oldham in 1962, suggests that there is a strong structural correlation (approximately 0.71) between the baseline values and change, even when ‘change’ is calculated on the basis of two columns of random numbers [59]. Mathematical coupling can lead to an artificially inflated association between initial value and change score when naïve methods are used [60]. The problem is that Bayesian methods, which are able to partially correct for this artefact to a significant degree, are not routinely applied in meta-analytic paper researches [64-66]. However, even these methods are not completely free from this phenomenon.

Taking into account that our data form a ‘star-shaped’ network, where all agents are compared to placebo effect, we employed a more advanced statistical method than other authors in the past, which is the NMA that is calculated for all treatments, the probability of being the best [31], and the SUCRA values [32]. In our case (star network pattern), NMA method relies only on the indirect comparison via placebo to contrast the different agents. In comparison, Huedo-Medina et al. [27] employed the naïve method of pooling the results, which has been criticised in meta-analysis bibliography that is liable to bias [53]. Conclusively, the results of the current paper suggesting that the use of Bayesian approach returns no role for initial severity should be considered to be strong. This finding is in accord with the conclusion other authors reached by analysing different data sets [67,68].

An important limitation in the Kirsch et al. data set is that it includes aggregate data rather than individual patient data. It has been recently shown that inference on patient-level characteristics, such as initial severity, using meta-regression models and aggregated evidence can be problematic due to aggregation bias [69]. As clearly stated in Additional file 2 (simple meta-regression in Section 3), this method has low power to detect any relationship when the number of studies is small.

A more complex issue which is beyond the scope of the current article is the intrinsic problems in the methodology of RCTs [70]. These problems tend to reduce the effect size for a number of reasons, with most prominent being the quality of recruited patients and the problems with the quantification of psychiatric symptoms, including the psychometric properties of the scales used. Even the concept of ‘severity’ is not satisfactorily studied. For example, some items like ‘depressed mood’ manifest a ceiling effect as severity grows while others like ‘suicidality’ manifest a floor effect as severity is reduced [71-81]. Both the HDRS and the MADRS describe a construct of depression which corresponds poorly to that defined by the DSM-IV and ICD-10 and include items corresponding to non-specific symptoms (e.g. sleep, appetite, anxiety; they might respond to a variety of non-antidepressant agents) or even side-effects (e.g. somatic symptoms) [77,78,82]. Also, it is obvious that the last observation carried forward method significantly contaminates efficacy with tolerability. However, no other results are usually available to analyse. Taking together that in many RCTs, agents like benzodiazepines are permitted in the placebo arm, the final score might not reflect the actual effect of the drug vs. placebo *per se* but somehow the add-on value of antidepressants on benzodiazepines. The RCTs are necessary for the licensing of drugs as safe and effective by the FDA, the EMEA, the MHRA, etc., but their usefulness should not be overstated, and their data should not be overused. Maybe it is time the raw data to be in the public domain, at least for products whose patent has expired. The way the lay press and especially the way medical scientists write for the lay press concerning antidepressants [83,84] cannot be considered in any other way but as being a reflection of a new type of stigma for depressed patients.

The results of the current study also suggest there is no ‘year’ effect; however, the changing severity of patients recruited over the years might result in a change in the observed difference between placebo and active drug. This is largely in accord with the conclusions of Undurraga and Baldessarini [9].

## Conclusion

The series of meta-analysis performed during the last decade made antidepressants maybe the best meta-analytically studied class of drugs in the whole of medicine. The results of the current analysis conclude the debate and suggest that antidepressants are clearly superior to placebo, and their efficacy is unrelated to initial severity. Thus, there is no scientific ground to deny mildly depressed patients the use of antidepressants, especially since they constitute the best validated treatment option for depression.

## Competing interests

KNF has received support concerning travel and accommodation expenses from various pharmaceutical companies in order to participate in medical congresses. He has also received honoraria for lectures from Astra-Zeneca, Janssen-Cilag, Eli-Lilly and a research grant from Pfizer Foundation. MS has received support concerning travel and accommodation expenses from various pharmaceutical companies. HJM has received grants or is a consultant for and on the speakership bureaus of AstraZeneca, Bristol-Myers Squibb, Eisai, Eli Lilly, GlaxoSmithKline, Janssen Cilag, Lundbeck, Merck, Novartis, Organon, Pfizer, Sanofi-Aventis, Schering-Plough, Schwabe, Sepracor, Servier and Wyeth. AAV has no competing interest.

## Authors’ contributions

KNF and HJM designed the study, wrote the first draft and the shaped the final draft. AAV made the analysis, wrote the additional files and interpreted the results. MS participated in writing all drafts and organized the material. All authors read and approved the final manuscript.

## Supplementary Material

Additional file 1 The complete set used in the current study. Click here for file

Additional file 2 The description, advantages and disadvantages of each of the methods. Click here for file

Additional file 3 The complete results of the analysis. Click here for file

## References

[B1] Turner EH Matthews AM Linardatos E Tell RA Rosenthal R Selective publication of antidepressant trials and its influence on apparent efficacy N Engl J Med 2008 358 3 252 260 10.1056/NEJMsa065779 18199864

[B2] Ghaemi SN Why antidepressants are not antidepressants: STEP-BD, STAR*D, and the return of neurotic depression Bipolar Disord 2008 10 8 957 968 10.1111/j.1399-5618.2008.00639.x 19594510

[B3] Bech P Cialdella P Haugh MC Birkett MA Hours A Boissel JP Tollefson GD Meta-analysis of randomised controlled trials of fluoxetine v. placebo and tricyclic antidepressants in the short-term treatment of major depression Br J Psychiatry 2000 176 421 428 10.1192/bjp.176.5.421 10912216

[B4] Moncrieff J Wessely S Hardy R Active placebos versus antidepressants for depression Cochrane Database Syst Rev 2004 1 CD003012 10.1002/14651858.CD003012.pub2 PMC8407353 14974002

[B5] Kirsch I Deacon BJ Huedo-Medina TB Scoboria A Moore TJ Johnson BT Initial severity and antidepressant benefits: a meta-analysis of data submitted to the Food and Drug Administration PLoS Med 2008 5 2 e45 10.1371/journal.pmed.0050045 18303940 PMC2253608

[B6] Fournier JC DeRubeis RJ Hollon SD Dimidjian S Amsterdam JD Shelton RC Fawcett J Antidepressant drug effects and depression severity: a patient-level meta-analysis JAMA 2010 303 1 47 53 10.1001/jama.2009.1943 20051569 PMC3712503

[B7] Barbui C Furukawa TA Cipriani A Effectiveness of paroxetine in the treatment of acute major depression in adults: a systematic re-examination of published and unpublished data from randomized trials CMAJ 2008 178 3 296 305 18227449 10.1503/cmaj.070693 PMC2211353

[B8] Khan A Leventhal RM Khan SR Brown WA Severity of depression and response to antidepressants and placebo: an analysis of the Food and Drug Administration database J Clin Psychopharmacol 2002 22 1 40 45 10.1097/00004714-200202000-00007 11799341

[B9] Undurraga J Baldessarini RJ Randomized, placebo-controlled trials of antidepressants for acute major depression: thirty-year meta-analytic review Neuropsychopharmacology 2012 37 4 851 864 10.1038/npp.2011.306 22169941 PMC3280655

[B10] Kirsch I Antidepressants and the placebo response Epidemiol Psichiatr Soc 2009 18 4 318 322 10.1017/S1121189X00000282 20170046

[B11] Kirsch I The Emperor’s New Drugs: Exploding the Antidepressant Myth 2009 London: The Bodley Head

[B12] Fountoulakis KN Hoschl C Kasper S Lopez-Ibor J Moller HJ The media and intellectuals’ response to medical publications: the anti-depressants’ case Ann Gen Psychiatry 2013 12 1 11 10.1186/1744-859X-12-11 23587303 PMC3643832

[B13] Cuijpers P Clignet F Van Meijel B Van Straten A Li J Andersson G Psychological treatment of depression in inpatients: a systematic review and meta-analysis Clin Psychol Rev 2011 31 3 353 360 10.1016/j.cpr.2011.01.002 21382540

[B14] Cuijpers P Smit F Bohlmeijer E Hollon SD Andersson G Efficacy of cognitive-behavioural therapy and other psychological treatments for adult depression: meta-analytic study of publication bias Br J Psychiatry 2010 196 3 173 178 10.1192/bjp.bp.109.066001 20194536

[B15] Cuijpers P Van Straten A Bohlmeijer E Hollon SD Andersson G The effects of psychotherapy for adult depression are overestimated: a meta-analysis of study quality and effect size Psychol Med 2009 40 2 211 223 19490745 10.1017/S0033291709006114

[B16] Driessen E Cuijpers P Hollon SD Dekker JJ Does pretreatment severity moderate the efficacy of psychological treatment of adult outpatient depression? A meta-analysis J Consult Clin Psychol 2010 78 5 668 680 20873902 10.1037/a0020570

[B17] Thase ME Larsen KG Kennedy SH Assessing the ‘true’ effect of active antidepressant therapy v. placebo in major depressive disorder: use of a mixture model Br J Psychiatry 2011 199 501 507 10.1192/bjp.bp.111.093336 22130749

[B18] Fountoulakis KN Moller HJ Efficacy of antidepressants: a re-analysis and re-interpretation of the Kirsch data Int J Neuropsychopharmacol 2011 14 3 405 412 10.1017/S1461145710000957 20800012

[B19] Horder J Matthews P Waldmann R Placebo, prozac and PLoS: significant lessons for psychopharmacology J Psychopharmacol 2011 25 10 1277 1288 10.1177/0269881110372544 20571143

[B20] Khan A Warner HA Brown WA Symptom reduction and suicide risk in patients treated with placebo in antidepressant clinical trials: an analysis of the Food and Drug Administration database Arch Gen Psychiatry 2000 57 4 311 317 10.1001/archpsyc.57.4.311 10768687

[B21] Barrett JE Williams JW Jr Oxman TE Frank E Katon W Sullivan M Hegel MT Cornell JE Sengupta AS Treatment of dysthymia and minor depression in primary care: a randomized trial in patients aged 18 to 59 years J Fam Pract 2001 50 5 405 412 11350703

[B22] DeRubeis RJ Hollon SD Amsterdam JD Shelton RC Young PR Salomon RM O’Reardon JP Lovett ML Gladis MM Brown LL Gallop R Cognitive therapy vs medications in the treatment of moderate to severe depression Arch Gen Psychiatry 2005 62 4 409 416 10.1001/archpsyc.62.4.409 15809408

[B23] Dimidjian S Hollon SD Dobson KS Schmaling KB Kohlenberg RJ Addis ME Gallop R McGlinchey JB Markley DK Gollan JK Atkins DC Dunner DL Randomized trial of behavioral activation, cognitive therapy, and antidepressant medication in the acute treatment of adults with major depression J Consult Clin Psychol 2006 74 4 658 670 16881773 10.1037/0022-006X.74.4.658

[B24] Elkin I Shea MT Watkins JT Imber SD Sotsky SM Collins JF Glass DR Pilkonis PA Leber WR Docherty JP National Institute of Mental Health Treatment of Depression Collaborative Research Program. General effectiveness of treatments Arch Gen Psychiatry 1989 46 11 971 982 discussion 983 10.1001/archpsyc.1989.01810110013002 2684085

[B25] Philipp M Kohnen R Hiller KO Hypericum extract versus imipramine or placebo in patients with moderate depression: randomised multicentre study of treatment for eight weeks BMJ 1999 319 7224 1534 1538 10.1136/bmj.319.7224.1534 10591711 PMC28296

[B26] Khan A Khan SR Leventhal RM Krishnan KR Gorman JM An application of the revised CONSORT standards to FDA summary reports of recently approved antidepressants and antipsychotics Biol Psychiatry 2002 52 1 62 67 10.1016/S0006-3223(02)01322-7 12079731

[B27] Huedo-Medina T Johnson B Kirsch I Kirsch et al. (2008) calculations are correct: reconsidering Fountoulakis & Moller’s re-analysis of the Kirsch data Int J Neuropsychopharmacol 2012 15 1193 1198 10.1017/S1461145711001878 22433169

[B28] Caldwell DM Ades AE Higgins JP Simultaneous comparison of multiple treatments: combining direct and indirect evidence BMJ 2005 331 7521 897 900 10.1136/bmj.331.7521.897 16223826 PMC1255806

[B29] Cooper NJ Peters J Lai MC Juni P Wandel S Palmer S Paulden M Conti S Welton NJ Abrams KR Bujkiewicz S Spiegelhalter D Sutton AJ How valuable are multiple treatment comparison methods in evidence-based health-care evaluation? Value Health 2011 14 2 371 380 10.1016/j.jval.2010.09.001 21296599

[B30] Mills EJ Ghement I O’Regan C Thorlund K Estimating the power of indirect comparisons: a simulation study PLoS One 2011 6 1 e16237 10.1371/journal.pone.0016237 21283698 PMC3025012

[B31] White IR Multivariate random-effects meta-regression: updates to mvmeta Stata Journal 2011 11 255 270

[B32] Salanti G Ades AE Ioannidis JP Graphical methods and numerical summaries for presenting results from multiple-treatment meta-analysis: an overview and tutorial J Clin Epidemiol 2011 64 2 163 171 10.1016/j.jclinepi.2010.03.016 20688472

[B33] Lambert P Eilers PH Bayesian proportional hazards model with time-varying regression coefficients: a penalized Poisson regression approach Stat Med 2005 24 24 3977 3989 10.1002/sim.2396 16320263

[B34] Lambert PC Sutton AJ Burton PR Abrams KR Jones DR How vague is vague? A simulation study of the impact of the use of vague prior distributions in MCMC using WinBUGS Stat Med 2005 24 15 2401 2428 10.1002/sim.2112 16015676

[B35] Friedrich JO Adhikari NK Beyene J Ratio of means for analyzing continuous outcomes in meta-analysis performed as well as mean difference methods J Clin Epidemiol 2011 64 5 556 564 10.1016/j.jclinepi.2010.09.016 21447428

[B36] Higgins J Green S Cochrane Handbook for Systematic Reviews of Interventions 2011 The Cochrane Collaboration http://www.cochrane-handbook.org

[B37] Sutton AJ Abrams KR Bayesian methods in meta-analysis and evidence synthesis Stat Methods Med Res 2001 10 4 277 303 10.1191/096228001678227794 11491414

[B38] Sharp SJ Thompson SG Analysing the relationship between treatment effect and underlying risk in meta-analysis: comparison and development of approaches Stat Med 2000 19 23 3251 3274 10.1002/1097-0258(20001215)19:23<3251::AID-SIM625>3.0.CO;2-2 11113958

[B39] Thompson SG Smith TC Sharp SJ Investigating underlying risk as a source of heterogeneity in meta-analysis Stat Med 1997 16 23 2741 2758 10.1002/(SICI)1097-0258(19971215)16:23<2741::AID-SIM703>3.0.CO;2-0 9421873

[B40] Salanti G Ades AE Ioannidis JP Graphical methods and numerical summaries for presenting results from multiple-treatment meta-analysis: an overview and tutorial J Clin Epidemiol 2010 64 2 163 171 20688472 10.1016/j.jclinepi.2010.03.016

[B41] Deeks JJ Issues in the selection of a summary statistic for meta-analysis of clinical trials with binary outcomes Stat Med 2002 21 11 1575 1600 10.1002/sim.1188 12111921

[B42] Engels EA Schmid CH Terrin N Olkin I Lau J Heterogeneity and statistical significance in meta-analysis: an empirical study of 125 meta-analyses Stat Med 2000 19 13 1707 1728 10.1002/1097-0258(20000715)19:13<1707::AID-SIM491>3.0.CO;2-P 10861773

[B43] Kirsch I Placebo psychotherapy: synonym or oxymoron? J Clin Psychol 2005 61 7 791 803 10.1002/jclp.20126 15827992

[B44] Kirsch I Conditioning, expectancy, and the placebo effect: comment on Stewart-Williams and Podd (2004) Psychol Bull 2004 130 2 341 343 discussion 344–345 14979776 10.1037/0033-2909.130.2.341

[B45] Kirsch I Johnson BT Moving beyond depression: how full is the glass? BMJ 2008 336 7645 629 630 18356211 10.1136/bmj.39520.483565.3A PMC2270982

[B46] Kirsch I Antidepressant drugs ‘work’ , but they are not clinically effective Br J Hosp Med (Lond) 2008 69 6 359 18646426

[B47] Kirsch I Challenging received wisdom: antidepressants and the placebo effect Mcgill J Med 2008 11 2 219 222 19148327 PMC2582668

[B48] Kirsch I Moncrieff J Clinical trials and the response rate illusion Contemp Clin Trials 2007 28 4 348 351 10.1016/j.cct.2006.10.012 17182286

[B49] Fountoulakis KN Moller HJ Antidepressant drugs and the response in the placebo group: the real problem lies in our understanding of the issue J Psychopharmacol 2011 26 5 744 750 21926425 10.1177/0269881111421969

[B50] Fountoulakis K Möller H Antidepressants vs. placebo: not merely a quantitative difference in response Int J Neuropsychopharmacol 2011 14 1435 1437 10.1017/S1461145711000964

[B51] Glenny AM Altman DG Song F Sakarovitch C Deeks JJ D’Amico R Bradburn M Eastwood AJ Indirect comparisons of competing interventions Health Technol Assess 2005 9 26 1 134 iii-iv 16014203 10.3310/hta9260

[B52] Song F Altman DG Glenny AM Deeks JJ Validity of indirect comparison for estimating efficacy of competing interventions: empirical evidence from published meta-analyses BMJ 2003 326 7387 472 10.1136/bmj.326.7387.472 12609941 PMC150178

[B53] Song F Loke YK Walsh T Glenny AM Eastwood AJ Altman DG Methodological problems in the use of indirect comparisons for evaluating healthcare interventions: survey of published systematic reviews BMJ 2009 338 b1147 10.1136/bmj.b1147 19346285 PMC2665205

[B54] Leucht S Hierl S Kissling W Dold M Davis JM Putting the efficacy of psychiatric and general medicine medication into perspective: review of meta-analyses Br J Psychiatry 2012 200 2 97 106 10.1192/bjp.bp.111.096594 22297588

[B55] Cohen J A power primer Psychol Bull 1992 112 1 155 159 19565683 10.1037//0033-2909.112.1.155

[B56] Tarr GP Glue P Herbison P Comparative efficacy and acceptability of mood stabilizer and second generation antipsychotic monotherapy for acute mania - a systematic review and meta-analysis J Affect Disord 2011 143 1–3 14 19 21145595 10.1016/j.jad.2010.11.009

[B57] Yildiz A Vieta E Leucht S Baldessarini RJ Efficacy of antimanic treatments: meta-analysis of randomized, controlled trials Neuropsychopharmacology 2011 36 2 375 389 10.1038/npp.2010.192 20980991 PMC3055677

[B58] Leucht S Arbter D Engel RR Kissling W Davis JM How effective are second-generation antipsychotic drugs? A meta-analysis of placebo-controlled trials Mol Psychiatry 2009 14 4 429 447 10.1038/sj.mp.4002136 18180760

[B59] Oldham P A note on the analysis of repeated measurements of the same subjects J Chronic Dis 1962 15 969 977 10.1016/0021-9681(62)90116-9 13939936

[B60] Tu YK Maddick IH Griffiths GS Gilthorpe MS Mathematical coupling can undermine the statistical assessment of clinical research: illustration from the treatment of guided tissue regeneration J Dent 2004 32 2 133 142 10.1016/j.jdent.2003.10.001 14749085

[B61] Cuijpers P Van Straten A Van Oppen P Andersson G Are psychological and pharmacologic interventions equally effective in the treatment of adult depressive disorders? A meta-analysis of comparative studies J Clin Psychiatry 2008 69 11 1675 1685 quiz 1839–1641 10.4088/JCP.v69n1102 18945396

[B62] Blomqvist N On the relation between change and initial value J Am Stat Assoc 1977 72 746 749

[B63] Jin P Toward a reconceptualization of the law of initial value Psychol Bull 1992 111 176 184 1539087 10.1037/0033-2909.111.1.176

[B64] Goodman SN Toward evidence-based medical statistics. 2: the Bayes factor Ann Intern Med 1999 130 12 1005 1013 10.7326/0003-4819-130-12-199906150-00019 10383350

[B65] Goodman SN Toward evidence-based medical statistics. 1: the P value fallacy Ann Intern Med 1999 130 12 995 1004 10.7326/0003-4819-130-12-199906150-00008 10383371

[B66] Johnson SR Tomlinson GA Hawker GA Granton JT Feldman BM Methods to elicit beliefs for Bayesian priors: a systematic review J Clin Epidemiol 2009 63 4 355 369 19716263 10.1016/j.jclinepi.2009.06.003

[B67] Gibbons RD Hur K Brown CH Davis JM Mann JJ Benefits from antidepressants: synthesis of 6-week patient-level outcomes from double-blind placebo-controlled randomized trials of fluoxetine and venlafaxine Arch Gen Psychiatry 2012 69 6 572 579 10.1001/archgenpsychiatry.2011.2044 22393205 PMC3371295

[B68] Melander H Salmonson T Abadie E Van Zwieten-Boot B A regulatory apologia–a review of placebo-controlled studies in regulatory submissions of new-generation antidepressants Eur Neuropsychopharmacol 2008 18 9 623 627 10.1016/j.euroneuro.2008.06.003 18621509

[B69] Petkova E Tarpey T Huang L Deng L Interpreting meta-regression: application to recent controversies in antidepressants’ efficacy Stat Med 2013 32 17 2875 2892 10.1002/sim.5766 23440635 PMC3800040

[B70] Turner EH Rosenthal R Efficacy of antidepressants BMJ 2008 336 7643 516 517 10.1136/bmj.39510.531597.80 18319297 PMC2265347

[B71] Bech P Rating scales for affective disorders: their validity and consistency Acta Psychiatr Scand Suppl 1981 295 1 101 7044046

[B72] Bech P Assessment scales for depression: the next 20 years Acta Psychiatr Scand Suppl 1983 310 117 130 6582768

[B73] Bech P The instrumental use of rating scales for depression Pharmacopsychiatry 1984 17 1 22 28 10.1055/s-2007-1017402 6709700

[B74] Bech P Rating scales in psychopharmacology. Statistical aspects Acta Psychiatr Belg 1988 88 4 291 302 3078609

[B75] Bech P Rating scales for mood disorders: applicability, consistency and construct validity Acta Psychiatr Scand Suppl 1988 345 45 55 3067540 10.1111/j.1600-0447.1988.tb08567.x

[B76] Bech P Psychometric developments of the Hamilton scales: the spectrum of depression, dysthymia, and anxiety Psychopharmacol Ser 1990 9 72 79 2204923 10.1007/978-3-642-75373-2_9

[B77] Bech P Modern psychometrics in clinimetrics: impact on clinical trials of antidepressants Psychother Psychosom 2004 73 3 134 138 10.1159/000076448 15031583

[B78] Bech P Rating scales in depression: limitations and pitfalls Dialogues Clin Neurosci 2006 8 2 207 215 16889106 10.31887/DCNS.2006.8.2/pbech PMC3181766

[B79] Bech P Applied psychometrics in clinical psychiatry: the pharmacopsychometric triangle Acta Psychiatr Scand 2009 120 5 400 409 10.1111/j.1600-0447.2009.01445.x 19807722

[B80] Bech P Allerup P Gram LF Reisby N Rosenberg R Jacobsen O Nagy A The Hamilton depression scale. Evaluation of objectivity using logistic models Acta Psychiatr Scand 1981 63 3 290 299 10.1111/j.1600-0447.1981.tb00676.x 7015793

[B81] Bech P Gram LF Dein E Jacobsen O Vitger J Bolwig TG Quantitative rating of depressive states Acta Psychiatr Scand 1975 51 3 161 170 10.1111/j.1600-0447.1975.tb00002.x 1136841

[B82] Bagby RM Ryder AG Schuller DR Marshall MB The Hamilton Depression Rating Scale: has the gold standard become a lead weight? Am J Psychiatry 2004 161 12 2163 2177 10.1176/appi.ajp.161.12.2163 15569884

[B83] The Epidemic of Mental Illness: Why? http://www.nybooks.com/articles/archives/2011/jun/23/epidemic-mental-illness-why/?pagination=false

[B84] The Illusions of Psychiatry http://www.nybooks.com/articles/archives/2011/jul/14/illusions-of-psychiatry/?pagination=false

