# Supplementary material for: No role for initial severity on the efficacy of antidepressants: results of a multi-meta-analysis
Source: Ann Gen Psychiatry. 2013 Aug 13;12:26. doi: 10.1186/1744-859X-12-26 (PMC3751863; doi:10.1186/1744-859X-12-26)
Supplement: Additional file 1 — The complete set used in the current study. [file 1744-859X-12-26-S1.docx]

| **company** | **ref** |  | **N** | **Baseline**  **drug** | **Change**  **drug** | **SD** | **N** | **Baseline**  **plc** | **Change**  **plc** | **SD** | **Publ year** |
| --- | --- | --- | --- | --- | --- | --- | --- | --- | --- | --- | --- |
| Fluoxetine(Eli Lilly and Company) | 19[27] | fluoxetine | 22 | 28.60 | 12.50 | 8.38 | 24 | 28.20 | 5.50 | 8.38 | 1985 |
|  | 25 | fluoxetine | 18 | 26.20 | 7.20 | 8.32 | 24 | 25.80 | 8.80 | 8.29 |  |
|  | 27[28] | fluoxetine | 181 | 27.50 | 11.00 | 9.53 | 163 | 28.20 | 8.40 | 9.53 | 1985 |
|  | 62(mild)[29] | fluoxetine | 299 | 17.00 | 5.89 | 5.78 | 56 | 17.40 | 5.82 | 5.48 | 1990 |
|  | 62(moderate) | fluoxetine | 297 | 24.30 | 8.82 | 7.79 | 48 | 24.30 | 5.69 | 7.79 |  |
| Venlafaxine(Wyeth Pharmaceuticals) | 203[30] | venlafaxine | 231 | 25.60 | 11.20 | 8.15 | 92 | 25.30 | 6.70 | 8.12 | 1998 |
|  | 206[31,36] | venlafaxine | 46 | 28.20 | 14.20 | 9.60 | 47 | 28.60 | 4.80 | 11.00 | 1995 |
|  | 301[31,32] | venlafaxine | 64 | 25.40 | 13.90 | 7.78 | 78 | 24.60 | 9.45 | 7.78 | 1994 |
|  | 302[33] | venlafaxine | 65 | 25.00 | 11.90 | 10.10 | 75 | 24.40 | 8.88 | 10.10 | 1994 |
|  | 303 | venlafaxine | 69 | 23.60 | 10.10 | 7.79 | 79 | 24.60 | 9.89 | 7.89 |  |
|  | 313[34,35] | venlafaxine | 227 | 25.70 | 11.00 | 8.18 | 75 | 25.40 | 9.49 | 8.16 | 1993 |
| Nefazodone(Bristol-MyersSquibb) | 030A2–0004/0005 | nefazodone | 74 | 23.40 | 10.00 | 7.55 | 70 | 24.00 | 9.84 | 7.69 |  |
|  | 030A2–0007[39] | nefazodone | 175 | 25.70 | 12.30 | 8.65 | 47 | 26.40 | 9.80 | 8.65 | 1990 |
|  | 03A0A-003[37] | nefazodone | 101 | 25.40 | 9.57 | 8.24 | 52 | 25.90 | 8.00 | 8.52 | 1994 |
|  | 03A0A-004A | nefazodone | 153 | 23.40 | 8.90 | 7.55 | 77 | 23.50 | 8.90 | 7.52 |  |
|  | 03A0A-004B[38] | nefazodone | 156 | 25.30 | 11.40 | 8.07 | 75 | 25.00 | 9.50 | 8.02 | 1995 |
|  | CN104–002 | nefazodone | 57 | 23.30 | 10.80 | 7.86 | 57 | 23.10 | 8.20 | 7.86 |  |
|  | CN104–005[40] | nefazodone | 86 | 24.50 | 12.00 | 7.85 | 90 | 23.30 | 8.00 | 7.85 | 1994 |
|  | CN104–006 | nefazodone | 80 | 23.80 | 10.00 | 7.37 | 78 | 23.50 | 8.90 | 7.37 |  |
| Paroxetine(GlaxoSmithKline) | 01–001 | paroxetine | 24 | 28.00 | 13.50 | 7.84 | 24 | 27.40 | 10.50 | 7.84 |  |
|  | 02–001[41,42] | paroxetine | 51 | 26.60 | 12.30 | 9.50 | 53 | 25.90 | 6.80 | 9.54 | 1989 |
|  | 02–002[43,44] | paroxetine | 36 | 25.00 | 10.90 | 8.64 | 34 | 24.90 | 5.80 | 8.63 | 1992 |
|  | 02–003[45] | paroxetine | 33 | 28.60 | 9.70 | 10.23 | 33 | 28.90 | 7.20 | 10.23 | 1992 |
|  | 02–004[46] | paroxetine | 36 | 28.90 | 12.70 | 6.66 | 38 | 27.30 | 7.60 | 6.66 | 1992 |
|  | 03–001[47,48] | paroxetine | 40 | 24.90 | 10.80 | 6.62 | 38 | 24.80 | 4.70 | 6.63 | 1989 |
|  | 03–002[49,50] | paroxetine | 40 | 24.90 | 8.00 | 6.89 | 40 | 25.60 | 6.20 | 6.89 | 1990 |
|  | 03–003 | paroxetine | 41 | 25.70 | 9.90 | 8.24 | 42 | 27.00 | 10.00 | 8.23 |  |
|  | 03–004[51] | paroxetine | 37 | 27.60 | 10.40 | 7.65 | 37 | 27.00 | 6.70 | 7.66 | 1992 |
|  | 03–005[52] | paroxetine | 40 | 26.10 | 10.00 | 9.87 | 42 | 26.80 | 4.10 | 9.85 | 1989 |
|  | 03–006[53] | paroxetine | 39 | 29.70 | 9.10 | 8.06 | 37 | 28.70 | 3.00 | 8.03 | 1992 |
|  | PAR07 | paroxetine | 13 | 30.50 | 13.10 | 10.22 | 12 | 28.30 | 10.90 | 10.22 |  |
|  | PAR09[54] | paroxetine | 403 | 25.20 | 9.10 | 7.10 | 51 | 24.50 | 8.20 | 7.10 | 1992 |
|  | UK06[55] | paroxetine | 19 | 23.70 | 6.00 | 5.90 | 22 | 24.20 | 6.20 | 7.20 | 1989 |
|  | UK09 | paroxetine | 20 | 26.80 | 8.80 | 10.50 | 21 | 25.50 | 4.50 | 8.80 |  |
|  | UK12 | paroxetine | 19 | 22.80 | 9.10 | 7.09 | 10 | 22.30 | 6.70 | 7.09 |  |
