# Supplementary material for: No role for initial severity on the efficacy of antidepressants: results of a multi-meta-analysis
Source: Ann Gen Psychiatry. 2013 Aug 13;12:26. doi: 10.1186/1744-859X-12-26 (PMC3751863; doi:10.1186/1744-859X-12-26)
Supplement: Additional file 2 — The description, advantages and disadvantages of each of the methods. [file 1744-859X-12-26-S2.docx]

**Webappendix 2**

# METHODS USED

## Effect Size : Raw Mean Difference (RMD)

## A.1. Assessment of publication bias

## Funnel Plot

## A.2. Frequentist Approach

## Simple random effects meta-analysis (simple REMA)

## Network random-effects meta-analysis (NMA)

## Simple random-effects meta-regression analysis (simple RE meta-regression) with one covariate: initial severity

## Simple RE meta-regression analysis with two covariates: initial severity and publication year

## A.3. Bayesian Approach

## Simple REMA

## NMA

## NMA random-effects meta-regression analysis

## NMA random-effects meta-regression analysis using 12 different prior distributions for the heterogeneity

## Simple RE meta-regression analysis with one covariate: initial severity

## Simple RE meta-regression analysis with one covariate: initial severity, using 12 different prior distributions for the heterogeneity

## Effect Size : Standardised Mean Difference (SMD)

## B.1. Assessment of publication bias

## Funnel Plot

## B.2. Frequentist Approach

## Simple REMA

## NMA

## Simple RE meta-regression analysis with one covariate: initial severity

## Simple RE meta-regression analysis with two covariates: initial severity and publication year

## B.3. Bayesian Approach

## Simple REMA

## NMA

## NMA RE meta-regression analysis

## NMA RE meta-regression analysis using 12 different prior distributions for the heterogeneity

## Simple RE meta-regression analysis with one covariate: initial severity

## Simple RE meta-regression analysis with one covariate: initial severity using 12 different prior distributions for the heterogeneity

# DESCRIPTION OF THE MODELS

Meta-analysis models can be viewed equivalently either as a special case of a weighted linear regression or as a hierarchical model. In a frequentist framework linear regression approaches are used (known also as ‘contrast-based’ models), whereas in a Bayesian implementation we use a hierarchical approach (known also as ‘arm-based’ models).

All frequentist approaches were implemented in STATA, whereas all Bayesian models in the freely available software WinBUGS 1.4.3^1^. For all Bayesian models two chains, after a burn-in period of 10000 Markov Chain Monte Carlo (MCMC) draws, were run until convergence. We used a visual inspection of the two Markov chains in the history plot to judge whether convergence was achieved.

## Simple random effects meta-analysis (REMA)

### Simple REMA ‘contrast-based’ model

Let $y_{i,TP}$ be the observed relative treatment effect of treatment T relative to placebo (P), e.g. raw mean difference (RMD) between the 2 groups, in study $i=1,..k$, with variance $v_{i,TP}$. Assuming $m_{i,P}$ and $m_{i,T}$ are the individual study means in placebo and treatment group respectively, ${sd}_{iP}$ and ${sd}_{iT}$ represent the standard deviation in each group and $n_{iP}$ and $n_{iT}$ are the respective sample sizes, then the RMD and its variance are obtained as

$$y_{i,TP}=m_{i,T}-m_{i,P}$$

$$v_{i,TP}=\frac{{sd}_{iP}^{2}}{n_{iP}}+\frac{{sd}_{iT}^{2}}{n_{iT}}$$

The model is structured under the assumption that the study variances, $v_{i,TP}$ are fixed and known. Under the random effects (RE) model the observed effect measures are modelled as

$$y_{i,TP}=\mu_{TP}+\delta_{i,TP}+\varepsilon_{i,TP}$$

$\varepsilon_{i,TP}\sim N(0,v_{i,TP})$, $\delta_{i,TP}\sim N(0,\tau_{TP}^{2})$

where $\mu_{TP}$ is the mean of the distribution of the underlying effects, $\delta_{i,TP}$ represent the random variation in the treatment effects across studies (RE) and $\varepsilon_{i,TP}$ is the random error in study $i=1,..k$. We set $\tau^{2}$ the between-study variability due to differences in the true effect sizes rather than chance, and we call it heterogeneity.

In the frequentist setting we use the inverse variance method, where the summary treatment effect $\mu_{TP}$ and its variance are estimated as

$$\mu_{TP}=\frac{\sum_{i=1}^{k} w_{i,TP}y_{i,TP}}{\sum_{i=1}^{k} w_{i,TP}}$$

$$Var(\mu_{TP})=\frac{1}{\sum_{i=1}^{k} w_{i,TP}}$$

with $w_{i,TP}=1/(v_{i,TP}+\tau^{2})$ representing the weight assigned to each study. We estimate $\tau^{2}$ using the DerSimonian and Laird (DL) estimator^2^. We fitted simple REMA in STATA using *metan*^3^ command.

### Simple REMA ‘arm-based’ model

Equivalently, under the random effects meta-analysis the observed treatment effect $y_{i,TP}$ is normally distributed with mean $\theta_{i,TP}$ and uncertainty reflected by the study variance $v_{i,TP}$.

$$y_{i,TP}\sim N(\theta_{i,TP},v_{i,TP})$$

Both linear regression and hierarchical models are equivalent as $\delta_{i,TP}$ is the difference between the mean $\mu_{TP}$ and the underlying study-specific mean $\theta_{i,TP}$.We assume that the true effects $\theta_{i,TP}$ vary between studies and are sampled from a normal distribution with expectation $\mu_{TP}$.

$$\theta_{i,TP}\sim N(\mu_{TP},\tau_{TP}^{2})$$

In the Bayesian framework we use the exact hierarchical model:

$$m_{iP}\sim N\left( \lambda_{ip},{{{sd}_{iP}^{2}}/n}_{iP} \right)$$

$$m_{iT}\sim N\left( \lambda_{iT},{{{sd}_{iT}^{2}}/n}_{iT} \right)$$

$$\lambda_{i,P}=u_{i}$$

$$\lambda_{i,T}=u_{i}+\theta_{i,TP}$$

$$\theta_{i,TP}\sim N\left( \mu_{TP},\tau_{TP}^{2} \right).$$

where $u_{i}$ is the mean of placebo from the baseline assumed to be normally distributed

$$u_{i}\sim N(m_{u},\sigma_{u}^{2})$$

We set the following prior distributions

$$\mu_{TP}\sim N(0,10000)$$

$$m_{u}\sim N(0,10000)$$

$$\tau_{TP}\sim N\left( 0,1 \right), \tau\geq0$$

$$\sigma_{u}\sim N\left( 0,1 \right), \tau\geq0$$

For standardised mean difference (SMD) effect measure we use the same model, where SMD is obtained as

$$y_{i,TP}=\frac{m_{i,T}-m_{i,P}}{sd_{i}^{pooled}}\cdot J_{i}$$

with pooled standard deviation $sd_{i}^{pooled}=\sqrt{\frac{(n_{i,P}-1)\cdot sd_{i,P}^{2}+(n_{i,T}-1)\cdot sd_{i,T}^{2}}{n_{i,P}+n_{i,T}-2}}$ and $J_{i}$ a correction factor^4^ for the overestimation of the real difference due to small sample sizes $J_{i}=1-\frac{3}{4(n_{i,P}+n_{i,T}-2)-1)}$.

## Simple random effects meta-regression

### Simple RE meta-regression ‘contrast-based’ model

We extend the previous model to include a study-level covariate $x_{i,TP}$ that represents initial severity as

$y_{i,TP}=\beta x_{i,TP}+\delta_{i,TP}+\varepsilon_{i,TP}$ (1)

$\varepsilon_{i,TP}\sim N(0,v_{i,TP})$, $\delta_{i,TP}\sim N(0,\tau_{TP}^{2})$

We estimate the between-study heterogeneity $\tau_{TP}^{2}$ using the DL^2^ method. We fitted simple meta-regression in STATA using *metareg*^5^ command. In the case where we model two covariates (initial severity $x_{i}$ and publication year $z_{i}$) formula (1) becomes

$$y_{i,TP}=\beta x_{i,TP}+\gamma z_{i,TP}+\delta_{i,TP}+\varepsilon_{i,TP}.$$

### Simple RE meta-regression ‘arm-based’ model

Equivalently, we extend the simple REMA hierarchical model as

$$y_{i,TP}\sim N(\theta_{i,TP},v_{i,TP})$$

$$\theta_{i,TP}\sim N(\beta x_{i,TP},\tau_{TP}^{2})$$

In the Bayesian setting the exact hierarchical model used is the following

$$\lambda_{i,P}=u_{i}$$

$$\lambda_{i,T}=u_{i}+\theta_{i,TP}^{*}$$

$$\theta_{i,TP}^{*}=\theta_{i,TP}+\beta x_{i,TP}$$

$$\theta_{i,TP}\sim N\left( \mu_{TP},\tau_{TP}^{2} \right)$$

We prefer though to centre the initial severity values around their mean, that is we subtract the mean initial severity ($\bar{x}_{TP}$) from each trial-specific covariate ($x_{i,TP}$), so as to improve the efficiency of the model estimation (‘correction’ for the ‘regression to the mean’ artefact):

$$\theta_{i,TP}^{*}=\theta_{i,TP}+\beta(x_{i,TP}-\bar{x}_{TP})$$

The parameters $\mu_{TP}$ and $u_{i}$ are given independent non-informative priors, whereas we set a weakly informative prior for $\tau$ as described previously. A vague prior is also assigned to $\beta$:

$$\beta\sim N(0,10000)$$

## Network random-effects meta-analysis (NMA) for the star-shaped network

### NMA ‘contrast-based’ model

Network meta-analysis can be viewed as a special case of multivariate meta-analysis. Consider for example a simple star-shaped network of evidence including three treatments $A, B, C$, and assume there are studies comparing $A$ versus $B$ and $A$ versus $C$ treatments, with common comparator $A$. Denoting by $y_{i,AB}$ and $y_{,iAC}$ the observed effect measures (e.g. RMD) $A$ versus $B$ and $A$ versus $C$, respectively, then each observed treatment effect is sampled from a normal distribution as

$$\left( \begin{aligned} y_{i,AB} \\ y_{i,AC} \end{aligned} \right)=\left( \begin{aligned} \mu_{AB} \\ \mu_{AC} \end{aligned} \right) +\left( \begin{aligned} \delta_{i,AB} \\ \delta_{i,AC} \end{aligned} \right)+\left( \begin{aligned} \varepsilon_{i,AB} \\ \varepsilon_{i,AC} \end{aligned} \right)$$

$$\left( \begin{aligned} \delta_{i,AB} \\ \delta_{i,AC} \end{aligned} \right)\sim N\left( \left( \begin{aligned} 0 \\ 0 \end{aligned} \right),\left( \begin{matrix} \tau^{2} & \tau^{2}/2 \\ \tau^{2}/2 & \tau^{2} \end{matrix} \right) \right)$$

$$\left( \begin{aligned} \varepsilon_{i,AB} \\ \varepsilon_{i,AC} \end{aligned} \right)\sim N\left( \left( \begin{aligned} 0 \\ 0 \end{aligned} \right),\left( \begin{matrix} v_{i,AB} & 0 \\ 0 & v_{i,AC} \end{matrix} \right) \right)$$

Then under the consistency assumption the estimated pooled effect size of treatment $B$ versus treatment $C$ is derived as

$${\mu_{BC}=\mu}_{AC}-\mu_{AB}$$

The model can be easily extended to more than three treatments. For further details see White et *al*^6^.

It should be noted that all comparisons in the network share a common $\tau^{2}$, which allows comparisons to ‘borrow strength’ from each other. In the frequentist setting we employ the model in STATA using the *mvmeta* command^7^ and we estimate a fixed $\tau^{2}$ using the restricted maximum likelihood (REML) estimator^8^. We also the probability that a treatment is the best (P(best)) for all antidepressants versus placebo comparisons^7^.

### NMA ‘arm-based’ model

Consider the previous simple star-shaped network of evidence. The observed treatment effect measures $y_{i,AB}$ and $y_{,iAC}$ are sampled from a normal distribution as

$$y_{i,AB}\sim N\left( \theta_{i,AB},v_{i,AB} \right), y_{i,AC}\sim N\left( \theta_{i,AC},v_{i,AC} \right)$$

and similarly for the random effects

$$\theta_{i,AB}\sim N\left( \mu_{AB},\tau^{2} \right), \theta_{i,AC}\sim N\left( \mu_{AC},\tau^{2} \right).$$

Under the consistency assumption ${\mu_{BC}=\mu}_{AC}-\mu_{AB}$.

The idea is extended to more than three treatments, where for any two treatments $j,k=\{A,B,C,D,E\}$ compared in study $i$ the model for a specific comparison $j$versus $k$ can be written as

$$y_{i,jk}\sim N\left( \theta_{i,jk},v_{i,jk} \right)$$

$$\theta_{i,jk}\sim N\left( \mu_{jk},\tau^{2} \right)$$

Setting $A$ the reference treatment and assuming consistency the means of the random-effects distributions are obtained as

$$\mu_{jk}=\mu_{Ak}-\mu_{Aj}$$

Note that all comparisons in the model share the same amount of heterogeneity. In the Bayesian framework $\tau^{2}$ is a random variable given a weakly informative prior distribution. We use the same prior distributions for $\mu_{\mathrm{jk}}$, $u_{i}$ and $\tau$ parameters as in simple REMA model. We also produced treatment ranking across all antidepressants versus placebo comparisons by estimating the surface under the cumulative ranking (SUCRA)^9^.

## NMA random-effects meta-regression

### NMA RE meta-regression ‘arm-based’ model

Extending the NMA hierarchical model to include a study-level covariate $x_{i,jk}$ that represents initial severity we use the following hierarchical model in a Bayesian setting

$$\lambda_{i,j}=u_{i}$$

$$\lambda_{i,k}=u_{i}+\theta_{i,jk}^{*}$$

$$\theta_{i,jk}^{*}=\theta_{i,jk}+\beta(x_{i,jk}-\bar{x}_{jk})$$

$$\theta_{i,jk}\sim N\left( \mu_{jk},\tau^{2} \right)$$

$$\mu_{jk}=\mu_{Ak}-\mu_{Aj}$$

We set the same prior distributions for $\mu_{jk}$, $u_{i}$, $\tau$ and $\beta$ as previously.

### Prior Distributions for $\boldsymbol{\tau}$ in NMA RE meta-regression

It has been shown that the choice of prior distribution is crucial, especially when few studies are included in the dataset^10^. We therefore employ 12 different priors in the NMA meta-regression model so as to evaluate any differences in the results. The following table shows the prior distributions we have set for the heterogeneity in the Bayesian model.

Table 1. Prior distributions for the heterogeneity.

| Prior 1 | $1/{\tau^{2}}\sim Pareto(1,0.001)$ |
| --- | --- |
| Prior 2 | $1/{\tau^{2}}\sim Pareto(1,0.25)$ |
| Prior 3 | $1/{\tau^{2}}\sim Gamma(0.01,0.01)$ |
| Prior 4 | $1/{\tau^{2}}\sim Gamma(0.1,0.1)$ |
| Prior 5 | $\tau\sim Uniform(0,100)$ |
| Prior 6 | $\tau\sim Uniform(0,2)$ |
| Prior 7 | $\tau\sim N(0,100)$*,* $\tau>0$ |
| Prior 8 | $\tau\sim N(0,1)$*,* $\tau>0$ |
| Prior 9 | $\tau^{2}\sim Uniform(0,1000)$ |
| Prior 10 | $\tau^{2}\sim Uniform(0,4)$ |
| Prior 11 | $log(\tau^{2})\sim Uniform(-10,10)$ |
| Prior 12 | $log(\tau^{2})\sim Uniform(-10,1.386)$ |

# ADVANTAGES AND LIMITATIONS

1. **Simple random effects meta-analysis**

Increases power and precision. Quantifies the treatments’ effectiveness and its uncertainty. Quantifies between-study heterogeneity. The validity depends on the quality of trials^4;11^.

1. **Network random effects meta-analysis**

Extension of Simple meta-analysis. Provides more powerful results by incorporating all evidence in the network^12;13^. Insights are provided when pairwise meta-analysis is not available. More specifically, in the case of a star-shaped network informed by AB, AC, AD comparisons, NMA uses all available study data to infer about the relative effectiveness of BC, BD, CD.

1. **Simple random effects meta-regression**

## The effects of multiple factors are investigated. We test whether there is a linear relationship between treatment effect and a covariate that differs across studies (e.g. initial severity)^14^. However, we should be aware of false-positive findings, i.e. finding a statistically significant result when there is no relationship in reality.

Comparing random-effects meta-analysis with random-effects meta-regression we determine how much heterogeneity is explained by the covariate. However, there is always the risk of confounding, i.e. a known or unknown covariate to be associated both with the covariate of interest and the treatment effect. We should be careful when investigating the relationship between treatment-effects and initial severity as they are inherently correlated. The Bayesian approach provides more reliable inferences than the frequentist one for this association by using an uninformative prior distribution for heterogeneity^15;16^. This method has low power to detect any relationship when the number of studies is small. There is also a potential for biases (e.g. aggregation bias)^17^.

1. **Network random effects meta-regression**

Extension of simple meta-regression analysis. The same characteristics as in NMA analysis. A difference in the results on these two models can be due to the adjustment of the covariate^18^.

1. **Bayesian approach in general**

Especially useful for small meta-analyses. Can assess robustness by using different priors. This method accounts for full uncertainty. However, the results depend on priors when few trials are available. It is possible to estimate the uncertainty of the heterogeneity in contrast to the frequentist approach. In the frequentist approach the heterogeneity parameter is assumed a known constant value, but in a Bayesian setting we set a prior distribution which allows us to infer about its (posterior) distribution. The heterogeneity uncertainty is always introduced in the results. When few studies are available the Bayesian estimation of heterogeneity may be problematic due to the choice of the prior distribution^10^. It is possible experts’ opinion to be introduced in the model.

1. **Frequentist approach in general**

Doesn’t estimate uncertainty for the heterogeneity. Difficult to estimate heterogeneity with few trials.

# REFERENCES

(1) Lunn DJ, Thomas A, Best N, Spiegelhalter D. WinBUGS - a Bayesian modelling framework: concepts, structure, and extensibility. *Statistics and Computing* 2000;325-337.

(2) DerSimonian R, Laird N. Meta-analysis in clinical trials. *Control Clin Trials* 1986;7:177-188.

(3) Harris R, Bradburn M, Deeks J, Harbord R, Altman D, Sterne J. metan: fixed- and random-effects meta-analysis. *Stata Journal* 2008;8:3-28.

(4) Borenstein M, Hedges LV, Higgins JPT, Rothstein HR. *Introduction to Meta-analysis* . 1st edition ed. Chichester, UK: John Wiley&Sons, 2009.

(5) Harbord R M, Higgins J P T. Meta-regression in Stata. *Stata Journal* 2008;8:493-519.

(6) White IR, Barret JK, Jackson D, Higgins JPT. Consistency and inconsistency in multiple treatments meta-analysis: model estimation using multivariate meta-regression. *Research Synthesis Methods* 2012;3:111-125.

(7) White IR. Multivariate random-effects meta-regression: Updates to mvmeta. *Stata Journal* 2011;11:255-270.

(8) Raudenbush S.W. Analyzing Effect Sizes: Random Effects Models. In: Cooper H., Hedges LV, Valentine J.C., eds. *The Handbook of Research Synthesis and Meta-Analysis*. 2nd edition ed. Russell Sage Foundation, New York; 2009;295-315.

(9) Salanti G, Ades AE, Ioannidis JP. Graphical methods and numerical summaries for presenting results from multiple-treatment meta-analysis: an overview and tutorial. *J Clin Epidemiol* 2011;64:163-171.

(10) Lambert PC, Sutton AJ, Burton PR, Abrams KR, Jones DR. How vague is vague? A simulation study of the impact of the use of vague prior distributions in MCMC using WinBUGS. *Stat Med* 2005;24:2401-2428.

(11) Higgins J., Green S. *Cochrane Handbook for Systematic Reviews of Interventions*. 2011.

(12) Salanti G, Higgins JP, Ades AE, Ioannidis JP. Evaluation of networks of randomized trials. *Stat Methods Med Res* 2008;17:279-301.

(13) Salanti G. Indirect and mixed-treatment comparison, network, or multiple-treatments meta-analysis: many names, many benefits, many concerns for the next generation evidence synthesis tool. *Research Synthesis Methods* 2012;3:80-97.

(14) Salanti G, Marinho V, Higgins JP. A case study of multiple-treatments meta-analysis demonstrates that covariates should be considered. *J Clin Epidemiol* 2009;62:857-864.

(15) Sutton AJ, Abrams KR. Bayesian methods in meta-analysis and evidence synthesis. *Stat Methods Med Res* 2001;10:277-303.

(16) Thompson SG, Smith TC, Sharp SJ. Investigating underlying risk as a source of heterogeneity in meta-analysis. *Stat Med* 1997;16:2741-2758.

(17) Petkova E, Tarpey T, Huang L, Deng L. Interpreting meta-regression: application to recent controversies in antidepressants' efficacy. *Stat Med* 2013.

(18) Salanti G, Dias S, Welton NJ et al. Evaluating novel agent effects in multiple-treatments meta-regression. *Stat Med* 2010;29:2369-2383.
