# Supplementary material for: No role for initial severity on the efficacy of antidepressants: results of a multi-meta-analysis
Source: Ann Gen Psychiatry. 2013 Aug 13;12:26. doi: 10.1186/1744-859X-12-26 (PMC3751863; doi:10.1186/1744-859X-12-26)
Supplement: Additional file 3 — The complete results of the analysis. [file 1744-859X-12-26-S3.docx]

# What is the bias in the Kirsch data set? How complete is this data set?

## Effect Size : Raw Mean Difference (RMD)

### Assessment of publication bias (Funnel plot)


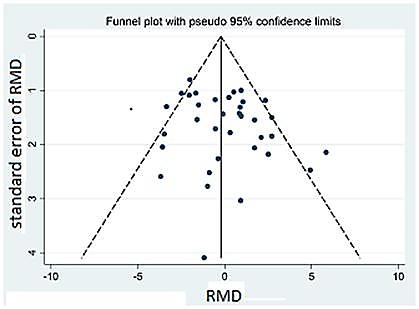


Figure 1. Funnel plot of the 35 trials

The visual inspection of the funnel plot in Figure 1 suggests that there is no asymmetry in the way the data points lie within the region defined by the two diagonal lines, which represent the 95% confidence limits around the summary treatment effect. Studies outside the 95% confidence limits are significant. There is no evidence for the presence of publication bias, as the funnel plot is visually symmetrical (Egger’s test p-value=0.133).

## Effect Size : Standardised Mean Difference (SMD)

### Assessment of publication bias (Funnel plot)


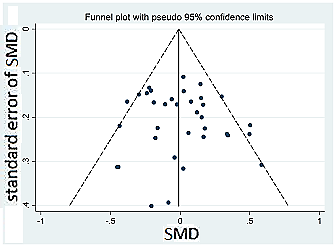


Figure 2. Funnel plot of the 35 trials

In Figure 2 a funnel plot is produced. Again, the data points lie symmetrically within the region defined by the two straight lines, which represent the 95% confidence limits around the summary treatment effect. The visual inspection of the funnel plot shows there is no evidence for the presence of publication bias, as the funnel plot is visually symmetrical (Egger’s test p-value=0.515).

# What is the heterogeneity of the studies in this data set?

## Effect Size : Raw Mean Difference (RMD)

Table 1. Heterogeneity ($\boldsymbol{\tau}^{\boldsymbol{2}}$) under the Frequentist and Bayesian methods when RMD is the effect size. In all Bayesian methods we assumed $\boldsymbol{\tau\sim N(0,1)}$*,* $\boldsymbol{\tau>0}$, whereas in all Frequentist methods we used the DerSimonian and Laird’s method except for the ΝΜΑ that we used the restricted maximum likelihood (REML). Under the Bayesian approach the posterior means and their Credible Intervals for $\boldsymbol{\tau}^{\boldsymbol{2}}$ are provided.

|  |  | Fluoxetine | Venlafaxine | Nefazodone | Paroxetine | Antidepressants |
| --- | --- | --- | --- | --- | --- | --- |
| Frequentist approach | Simple RE meta-analysis (DL) | 3.53 | 4.79 | 0.40 | 2.03 | 2.50 |
|  | ΝΜΑ (REML) |  |  |  |  | 2.31 |
|  | Simple RE meta-regression analysis (DL) |  |  |  |  | 1.57 |
|  | Simple RE meta-regression analysis (DL)  (outlier excluded) |  |  |  |  | 1.68 |
| Bayesian approach | Simple RE meta-analysis | 1.85  (0.10, 6.55) | 0.94  (0.01, 4.71) | 0.77  (0.01, 3.27) | 0.92  (0.01, 3.72) | 1.61  (0.53, 3.53) |
|  | ΝΜΑ |  |  |  |  | 0.71  (0.24, 0.98) |
|  | ΝΜΑ RE meta-regression analysis |  |  |  |  | 0.59  (0.01, 2.19) |
|  | ΝΜΑ RE meta-regression analysis  (outlier excluded) |  |  |  |  | 0.64  (0.01, 2.34) |
|  | Simple RE meta-regression analysis |  |  |  |  | 0.58  (0.00,2.13) |
|  | Simple RE meta-regression analysis  (outlier excluded) |  |  |  |  | 0.62  (0.00, 2.28) |

Under the RMD and the Simple RE meta-analysis in a frequentist framework a large amount of the heterogeneity ($\tau^{2}$) is produced in comparison to the Bayesian method. The larger amount of heterogeneity under the frequentist methods rather than the Bayesian methods might be explained by the presence of correlation between initial severity and RMD. This association is investigated by applying the meta-regression approach. In Simple RE meta-regression method we observe that this covariate explains some of the variability among the treatment effects between-studies (the amount of the heterogeneity is reduced). However, under the frequentist approach it is possible to erroneously observe the relationship between initial severity and treatment effectiveness due to the statistical artefact ‘regression to the mean’^1^. In the Bayesian framework this problem is addressed relaxing the strong relationship between RMD and initial severity (see section 5).

Network meta-analysis (NMA) relies on the randomization of RCTs in direct comparison, as the ordinary meta-analysis requires, but provides more precise estimates. The NMA methods require the similarity and consistency assumptions in order to provide meaningful results. Under the similarity assumption the treatment effect estimates are similar in factors that could affect the relative treatment effects. In our dataset the consistency assumption cannot be evaluated due to the shape of the network.

We repeated the analysis for 12 different priors for the heterogeneity according to Lambert et *al*^5^. We observe that the magnitude of the heterogeneity differs according to the prior we use. Since the number of studies within the four comparisons is small to moderate, the choice of the prior distribution can lead to a marked variation of the results.

Table 2. Heterogeneity ($\boldsymbol{\tau}^{\boldsymbol{2}}$) estimated under the Simple RE meta-regression and ΝΜΑ RE meta-regression analysis in a Bayesian framework, when 12 different priors were applied. The posterior means and their Credible Intervals for $\boldsymbol{\tau}^{\boldsymbol{2}}$ are provided. The RMD is considered as the applying effect size.

| Prior Distribution for heterogeneity | Simple RE meta-regression | ΝΜΑ RE meta-regression |
| --- | --- | --- |
| $1/{\tau^{2}}\sim Pareto(1,0.001)$ | 1.12 (0.14,3.24) | 1.19 (0.15,3.57) |
| $1/{\tau^{2}}\sim Pareto(1,0.25)$ | 1.08 (0.14,3.03) | 1.14 (0.15,3.20) |
| $1/{\tau^{2}}\sim Gamma(0.01,0.01)$ | 0.49 (0.01,2.19) | 0.49 (0.01,2.31) |
| $1/{\tau^{2}}\sim Gamma(0.1,0.1)$ | 0.66 (0.07,2.28) | 0.67 (0.07,2.43) |
| $\tau\sim Uniform(0,100)$ | 0.76 (0.01,2.72) | 0.81 (0.01,2.96) |
| $\tau\sim Uniform(0,2)$ | 0.76 (0.01,2.62) | 0.79 (0.01,2.79) |
| $\tau\sim N(0,100)$*,* $\tau>0$ | 0.76 (0.01,2.69) | 0.79 (0.01,2.92) |
| $\tau\sim N(0,1)$*,* $\tau>0$ | 0.58 (0.00,2.13) | 0.59 (0.01,2.19) |
| $\tau^{2}\sim Uniform(0,1000)$ | 1.14 (0.14,3.28) | 1.29 (0.14,3.65) |
| $\tau^{2}\sim Uniform(0,4)$ | 1.08 (0.14,3.06) | 1.14 (0.14,3.24) |
| $log(\tau^{2})\sim Uniform(-10,10)$ | 0.24 (0.00,1.99) | 0.25 (0.00,2.10) |
| $log(\tau^{2})\sim Uniform(-10,1.386)$ | 0.24 (0.00,1.96) | 0.24 (0.00,2.04) |

## Effect Size : Standardised Mean Difference (SMD)

Empirical evidence has shown that RMD is more heterogeneous than SMD, which agrees with our analysis^6^. When the SMD is applied the amount of heterogeneity is considerably lower than under the use of RMD. It is advisable that a meta-analysis should be conducted by using the method that is associated with the least heterogeneity^7^. Erroneous choices about the analysis of the results or an inappropriate choice of the effect measure may exacerbate the extent of heterogeneity^6,8,9^. However, the decreased heterogeneity in SMD rather than RMD could be due to a possible bias especially in small studies^6^.

Table 3. Heterogeneity ($\boldsymbol{\tau}^{\boldsymbol{2}}$) under the Frequentist and Bayesian methods. In all Bayesian methods we assumed $\boldsymbol{\tau\sim N(0,1)}$*,* $\boldsymbol{\tau>0}$, whereas in all Frequentist methods we used the DerSimonian and Laird’s method except for the ΝΜΑ that we used the restricted maximum likelihood (REML). Under the Bayesian approach the posterior means and their Credible Intervals for $\boldsymbol{\tau}^{\boldsymbol{2}}$ are provided. The SMD is considered as the applying effect size.

|  |  | Fluoxetine | Venlafaxine | Nefazodone | Paroxetine | Antidepressants |
| --- | --- | --- | --- | --- | --- | --- |
| Frequentist approach | Simple RE meta-analysis (DL) | 0.04 | 0.06 | 0.01 | 0.03 | 0.03 |
|  | ΝΜΑ (REML) |  |  |  |  | 0.03 |
|  | Simple RE meta-regression analysis (DL) |  |  |  |  | 0.02 |
|  | Simple RE meta-regression analysis (DL) (outlier excluded) |  |  |  |  | 0.02 |
| Bayesian approach | Simple RE meta-analysis | 0.03 (0.00,0.38) | 0.07 (0.00,0.44) | 0.01 (0.00,0.07) | 0.02 (0.00,0.11) | 0.02 (0.00,0.05) |
|  | ΝΜΑ |  |  |  |  | 0.01 (0.00,0.05) |
|  | ΝΜΑ RE meta-regression analysis |  |  |  |  | 0.01 (0.00,0.05) |
|  | ΝΜΑ RE meta-regression analysis (outlier excluded) |  |  |  |  | 0.01 (0.00,0.05) |
|  | Simple RE meta-regression analysis |  |  |  |  | 0.01 (0.00,0.05) |
|  | Simple RE meta-regression analysis (outlier excluded) |  |  |  |  | 0.01 (0.00,0.05) |

Table 4. Heterogeneity ($\boldsymbol{\tau}^{\boldsymbol{2}}$) estimated under the Simple RE meta-regression and ΝΜΑ RE meta-regression analysis in a Bayesian framework, when 12 different priors were applied. The posterior means and their Credible Intervals for $\boldsymbol{\tau}^{\boldsymbol{2}}$ are provided. The SMD is considered as the applying effect size.

| Prior Distribution for heterogeneity | Simple RE meta-regression | ΝΜΑ RE meta-regression |
| --- | --- | --- |
| $1/{\tau^{2}}\sim Pareto(1,0.001)$ | 0.02 (0.00,0.05) | 0.02 (0.00,0.06) |
| $1/{\tau^{2}}\sim Pareto(1,0.25)$ | 0.02 (0.00,0.05) | 0.02 (0.00,0.06) |
| $1/{\tau^{2}}\sim Gamma(0.01,0.01)$ | 0.02 (0.00,0.04) | 0.02 (0.00,0.05) |
| $1/{\tau^{2}}\sim Gamma(0.1,0.1)$ | 0.04 (0.02,0.07) | 0.04 (0.02,0.08) |
| $\tau\sim Uniform(0,100)$ | 0.01 (0.00,0.04) | 0.01 (0.00,0.05) |
| $\tau\sim Uniform(0,2)$ | 0.01 (0.00,0.04) | 0.01 (0.00,0.05) |
| $\tau\sim N(0,100)$, $\tau>0$ | 0.01 (0.00,0.04) | 0.01 (0.00,0.05) |
| $\tau\sim N(0,1)$, $\tau>0$ | 0.01 (0.00,0.05) | 0.01 (0.00,0.05) |
| $\tau^{2}\sim Uniform(0,1000)$ | 0.02 (0.00,0.05) | 0.02 (0.00,0.06) |
| $\tau^{2}\sim Uniform(0,4)$ | 0.02 (0.00,0.05) | 0.02 (0.00,0.06) |
| $log(\tau^{2})\sim Uniform(-10,10)$ | 0.00 (0.00,0.03) | 0.00 (0.00,0.04) |
| $log(\tau^{2})\sim Uniform(-10,1.386)$ | 0.01 (0.00,0.03) | 0.01 (0.00,0.04) |

The estimate of the heterogeneity for the Bayesian approach is smaller than the frequentist one. Since the Bayesian model treats the heterogeneity parameter as a ‘varying’ parameter rather than constant a credible interval (CrI) for the heterogeneity is constructed. The 95% CrI for the heterogeneity reflects the uncertainty surrounding this parameter. Since the number of studies being combined in each head-to-head meta-analysis is relatively small the magnitude of the uncertainty of $\tau^{2}$ is expected to be quite large.

# and 5. What is the efficacy of antidepressants vs. placebo (effect size)?

## Effect Size : Raw Mean Difference (RMD)

All methods suggest that Venlafaxine and Paroxetine are the most efficient interventions among the four, except for the ΝΜΑ RE meta-regression analysis when adjusting for RMD that suggest Venlafaxine and Nefazodone. The ΝΜΑ (unadjusted RMD) suggests that Fluoxetine has very low efficacy compared to the rest of the interventions and Paroxetine is the second most efficient treatment. However, the ΝΜΑ RE meta-regression analysis suggests that Nefazodone is the second best treatment and that Fluoxetine does not differ significantly from Paroxetine. Comparing these findings we conclude that the inclusion of the initial severity in the model affects significantly the results.

We repeated the analysis for 12 different priors for the heterogeneity according to Lambert et *al*^5^. We observe that all 12 analyses suggest that Venlafaxine is the most efficient antidepressant among the four.

Table 5. Summary treatment effects when applying RMD along with their 95% Confidence Intervals under the Frequentist methods or their 95% Credible Intervals under the Bayesian methods.

|  | Frequentist Approach | | Bayesian Approach | | | |
| --- | --- | --- | --- | --- | --- | --- |
| Treatment | Simple RE meta-analysis | ΝΜΑ | Simple RE meta-analysis | Simple RE meta-regression | ΝΜΑ  (unadjusted RMD) | ΝΜΑ RE meta-regression analysis  (adjusted RMD) |
| Fluoxetine | 2.07  (-0.04, 4.19) | 2.02  (0.17, 3.87) | 1.91  (0.16, 3.8) |  | 1.34  (0.11, 2.59) | 2.16  (0.94, 3.36) |
| Venlafaxine | 3.55  (1.47, 5.64) | 3.44  (1.79, 5.09) | 3.23  (1.87, 4.71) |  | 3.69  (2.54, 4.85) | 3.73  (2.69, 4.80) |
| Nefazodone | 1.65  (0.68, 2.62) | 1.69  (0.32, 3.06) | 1.73  (0.62, 2.84) |  | 2.33  (1.37, 3.29) | 2.75  (1.84, 3.66) |
| Paroxetine | 3.38  (2.19, 4.57) | 3.38  (2.16, 4.61) | 3.32  (2.26, 4.43) |  | 2.60  (1.69, 3.53) | 2.39  (1.52, 3.27) |
| Antidepressants | 2.71  (1.96, 3.45) |  | 2.61  (1.94, 3.30) | 2.77  (2.18, 3.36) |  |  |
| Antidepressants (outlier excluded) |  |  |  | 2.82  (2.21, 3.44) |  |  |

Table 6. RMD along with its 95% Credible Interval under the ΝΜΑ RE meta-regression and Simple RE meta-regression methods in a Bayesian framework. Twelve different priors for $\boldsymbol{\tau}^{\mathbf{2}}$ are considered.

|  | ΝΜΑ RE meta-regression | | | | Simple RE meta-regression |
| --- | --- | --- | --- | --- | --- |
| Prior Distribution for heterogeneity | Fluoxetine | Venlafaxine | Nefazodone | Paroxetine | Antidepressants |
| $1/{\tau^{2}}\sim Pareto(1,0.001)$ | 2.31  (0.73, 3.89) | 3.70  (2.35, 5.08) | 2.56  (1.37, 3.75) | 2.62  (1.57, 3.70) | 2.78  (2.14, 3.43) |
| $1/{\tau^{2}}\sim Pareto(1,0.25)$ | 2.30  (0.75, 3.87) | 3.70  (2.36, 5.06) | 2.56  (1.38, 3.74) | 2.62  (1.57, 3.69) | 2.78  (2.15, 3.42) |
| $1/{\tau^{2}}\sim Gamma(0.01,0.01)$ | 2.26  (0.93, 3.61) | 3.69  (2.53, 4.86) | 2.61  (1.59, 3.63) | 2.53  (1.58, 3.50) | 2.77  (2.18, 3.36) |
| $1/{\tau^{2}}\sim Gamma(0.1,0.1)$ | 2.26  (0.88, 3.66) | 3.70  (2.50, 4.92) | 2.61  (1.56, 3.67) | 2.54  (1.57, 3.53) | 2.76  (2.19, 3.34) |
| $\tau\sim Uniform(0,100)$ | 2.29  (0.83, 3.75) | 3.70  (2.45, 4.99) | 2.59  (1.48, 3.70) | 2.58  (1.58, 3.61) | 2.77  (2.17, 3.39) |
| $\tau\sim Uniform(0,2)$ | 2.29  (0.84, 3.74) | 3.70  (2.45, 4.97) | 2.59  (1.49, 3.69) | 2.58  (1.59, 3.60) | 2.77  (2.17, 3.39) |
| $\tau\sim N(0,100)$*,* $\tau>0$ | 2.28  (0.83, 3.74) | 3.69  (2.44, 4.98) | 2.58  (1.48, 3.68) | 2.58  (1.58, 3.61) | 2.77  (2.17, 3.38) |
| $\tau\sim N(0,1)$*,* $\tau>0$ | 2.26  (0.90, 3.63) | 3.70  (2.53, 4.90) | 2.61  (1.58, 3.65) | 2.53  (1.58, 3.51) | 2.77  (2.18, 3.36) |
| $\tau^{2}\sim Uniform(0,1000)$ | 2.29  (0.70, 3.89) | 3.70  (2.33, 5.11) | 2.54  (1.34, 3.75) | 2.64  (1.57, 3.73) | 2.78  (2.14, 3.43) |
| $\tau^{2}\sim Uniform(0,4)$ | 2.31  (0.74, 3.87) | 3.70  (2.36, 5.08) | 2.56  (1.39, 3.74) | 2.62  (1.57, 3.69) | 2.78  (2.15, 3.43) |
| $log(\tau^{2})\sim Uniform(-10,10)$ | 2.28  (1.02, 3.57) | 3.66  (2.57, 4.78) | 2.61  (1.65, 3.58) | 2.50  (1.59, 3.44) | 2.75  (2.19, 3.31) |
| $log(\tau^{2})\sim Uniform(-10,1.386)$ | 2.28  (1.03, 3.55) | 3.67  (2.58, 4.77) | 2.60  (1.64, 3.57) | 2.51  (1.59, 3.47) | 2.75  (2.20, 3.31) |

## Effect Size : Standardised Mean Difference (SMD)

Both ΝΜΑ (unadjusted SMD) and ΝΜΑ RE meta-regression analysis (adjusted SMD) agree that Venlafaxine and Paroxetine are the most efficient interventions among the four. Comparing these findings we conclude that the inclusion of the initial severity in the model does not affect significantly the results. The uncertainty associated with the heterogeneity parameter under the Bayesian methods can lead to a wider Confidence Interval (CI) for the pooled treatment effect than the Frequentist ones.

Again repeating the analysis for 12 different priors for the heterogeneity we observe that Venlafaxine is the most efficient antidepressant among the four.

Table 7. Summary treatment effects when applying SMD along with their 95% Confidence Intervals under the Frequentist methods or their 95% Credible Intervals under the Bayesian methods.

|  | Frequentist Approach | | Bayesian Approach | | | |
| --- | --- | --- | --- | --- | --- | --- |
| Treatment | Simple RE meta-analysis | ΝΜΑ | Simple RE meta-analysis | Simple RE meta-regression | ΝΜΑ  (unadjusted SMD) | ΝΜΑ RE meta-regression analysis  (adjusted SMD) |
| Fluoxetine | 0.25  (0.01, 0.49) | 0.25  (0.03, 0.47) | 0.24  (-0.02, 0.52) |  | 0.22  (0.04, 0.41) | 0.26  (0.08, 0.43) |
| Venlafaxine | 0.41  (0.18, 0.63) | 0.40  (0.21, 0.59) | 0.40  (0.12, 0.69) |  | 0.41  (0.24, 0.57) | 0.41  (0.25, 0.56) |
| Nefazodone | 0.21  (0.09, 0.33) | 0.21  (0.05, 0.37) | 0.21  (0.07, 0.35) |  | 0.27  (0.13, 0.41) | 0.29  (0.15, 0.43) |
| Paroxetine | 0.42  (0.28, 0.57) | 0.42  (0.28, 0.57) | 0.42  (0.28, 0.57) |  | 0.37  (0.24, 0.50) | 0.35  (0.23, 0.48) |
| Antidepressants | 0.33  (0.24, 0.42) |  | 0.32  (0.25, 0.40) | 0.34  (0.27, 0.42) |  |  |
| Antidepresants  (excluding outlier) |  |  |  | 0.34  (0.27, 0.42) |  |  |

Table 8. SMD along with its 95% Credible Interval under the ΝΜΑ RE meta-regression and Simple RE meta-regression methods in a Bayesian framework. Twelve different priors for $\boldsymbol{\tau}^{\mathbf{2}}$ are considered.

|  | ΝΜΑ RE meta-regression | | | | Simple RE meta-regression |
| --- | --- | --- | --- | --- | --- |
| Prior Distribution for heterogeneity | Fluoxetine | Venlafaxine | Nefazodone | Paroxetine | Antidepressants |
| $1/{\tau^{2}}\sim Pareto(1,0.001)$ | 0.26  (0.06, 0.46) | 0.41  (0.24, 0.57) | 0.29  (0.14, 0.44) | 0.36  (0.22, 0.49) | 0.33  (0.26, 0.41) |
| $1/{\tau^{2}}\sim Pareto(1,0.25)$ | 0.26  (0.06, 0.46) | 0.41  (0.24, 0.57) | 0.29  (0.14, 0.44) | 0.36  (0.22, 0.49) | 0.33  (0.26, 0.41) |
| $1/{\tau^{2}}\sim Gamma(0.01,0.01)$ | 0.26  (0.07, 0.45) | 0.41  (0.07, 0.57) | 0.29  (0.15, 0.44) | 0.35  (0.22, 0.49) | 0.33  (0.26, 0.41) |
| $1/{\tau^{2}}\sim Gamma(0.1,0.1)$ | 0.26  (0.03, 0.50) | 0.41  (0.21, 0.61) | 0.29  (0.11, 0.47) | 0.36  (0.21, 0.51) | 0.34  (0.25, 0.43) |
| $\tau\sim Uniform(0,100)$ | 0.26  (0.08, 0.44) | 0.41  (0.25, 0.56) | 0.29  (0.15, 0.43) | 0.35  (0.23, 0.48) | 0.33  (0.26, 0.41) |
| $\tau\sim Uniform(0,2)$ | 0.26  (0.08, 0.44) | 0.41  (0.25, 0.56) | 0.29  (0.15, 0.43) | 0.36  (0.23, 0.48) | 0.33  (0.26, 0.41) |
| $\tau\sim N(0,100)$*,* $\tau>0$ | 0.26  (0.08, 0.43) | 0.41  (0.25, 0.56) | 0.29  (0.15, 0.43) | 0.35  (0.23, 0.48) | 0.34  (0.27, 0.42) |
| $\tau\sim N(0,1)$*,* $\tau>0$ | 0.26  (0.08, 0.44) | 0.41  (0.25, 0.56) | 0.29  (0.15, 0.43) | 0.35  (0.23, 0.48) | 0.33  (0.25, 0.41) |
| $\tau^{2}\sim Uniform(0,1000)$ | 0.26  (0.06, 0.46) | 0.41  (0.24, 0.58) | 0.29  (0.14, 0.44) | 0.36  (0.22, 0.49) | 0.33  (0.26, 0.41) |
| $\tau^{2}\sim Uniform(0,4)$ | 0.26  (0.06, 0.46) | 0.41  (0.24, 0.58) | 0.29  (0.14, 0.44) | 0.36  (0.22, 0.49) | 0.33  (0.26, 0.42) |
| $log(\tau^{2})\sim Uniform(-10,10)$ | 0.25  (0.09, 0.41) | 0.41  (0.27, 0.54) | 0.29  (0.17, 0.42) | 0.35  (0.23, 0.47) | 0.33  (0.26, 0.40) |
| $log(\tau^{2})\sim Uniform(-10,1.386)$ | 0.25  (0.09, 0.42) | 0.41  (0.27, 0.55) | 0.30  (0.17, 0.42) | 0.35  (0.23, 0.47) | 0.33  (0.26, 0.40) |

# Are all antidepressants equal in terms of efficacy?

## Effect Size : Raw Mean Difference (RMD)

Table 9. Efficacy of all antidepressant drugs according to ΝΜΑ (light blue cells) and ΝΜΑ RE meta-regression analysis (blue cells). In dark blue cells the ΝΜΑ RE meta-regression analysis’ results are reported after the exclusion of the outlying study. Drugs are reported in order according to efficacy ranking. Comparisons between treatments should be read from left to right, where a negative value ($\boldsymbol{RMD<0}$) favours the column-defining treatment. Significant results are in bold.

|  | | **ΝΜΑ** | | | | |
| --- | --- | --- | --- | --- | --- | --- |
| **ΝΜΑ RE meta-regression analysis** | **(outlier excluded)** | **Placebo** | **-1.34**  **(-2.59, -0.11)** | **-3.69**  **(-4.85,-2.54)** | **-2.33**  **(-3.29,-1.37)** | **-2.60**  **(-3.53,-1.69)** |
|  |  | **-2.16**  **(-3.36, -0.94)** | **Fluoxetine** | **-2.35**  **(-4.00,-0.69)** | -0.99  (-2.52, 0.56) | -1.27  (-2.74, 0.22) |
|  |  | **-2.02**  **(-3.41, -0.63)** |  |  |  |  |
|  |  | **-3.73**  **(-4.80,-2.69)** | **-1.58**  **(-3.14,-0.04)** | **Venlafaxine** | 1.36  (-0.09, 2.82) | 1.84  (-0.34, 2.50) |
|  |  | **-3.82**  **(-4.90,-2.76)** | **-1.80**  **(-3.54,-0.08)** |  |  |  |
|  |  | **-2.75**  **(-3.66,-1.84)** | -0.59  (-2.01, 0.82) | 0.99  (-0.32, 2.32) | **Nefazodone** | -0.28  (-1.56, 1.00) |
|  |  | **-2.89**  **(-3.87,-1.90)** | -0.87  (-2.58, 0.84) | 0.93  (-0.40, 2.30) |  |  |
|  |  | **-2.39**  **(-3.27,-1.52)** | -0.23  (-1.69, 1.21) | 1.35  (-0.03, 2.66) | 0.36  (-0.87, 1.57) | **Paroxetine** |
|  |  | **-2.39**  **(-3.28,-1.51)** | -0.37  (-1.95, 1.18) | 1.43  (-0.10, 2.76) | 0.50  (-0.79, 1.77) |  |

NMA methods provide not only more powerful results by incorporating evidence from both direct and indirect comparisons, but also provide insights when there are no trials directly comparing the treatments of interest. In case of a star network, like our dataset, all antidepressants are compared only to placebo with no available comparisons between them. In such a case ΝΜΑ compares antidepressants indirectly.

Table 10. Order of treatments according to the P(best) under the frequentist approach and SUCRA Values along with their Credible Intervals (CrI) under the Bayesian approach. P(Best) was computed from 10000 parametric bootstrap samples.

|  | Frequentist Approach  (P(best)) | Bayesian Approach  (SUCRA Values) | |
| --- | --- | --- | --- |
| Treatment | ΝΜΑ | ΝΜΑ  (CrI) | ΝΜΑ RE meta-regression analysis  (CrI) |
| Placebo | 0.00 | 0.00 (0.00, 0.00) | 0.00 (0.00, 0.00) |
| Fluoxetine | 0.05 | 0.28 (0.25, 0.75) | 0.40 (0.25, 0.75) |
| Venlafaxine | 0.50 | 0.97 (0.75, 1.00) | 0.97 (0.75, 1.00) |
| Nefazodone | 0.01 | 0.57 (0.25, 0.75) | 0.65 (0.25, 1.00) |
| Paroxetine | 0.44 | 0.67 (0.25, 1.00) | 0.48 (0.25, 0.75) |

According to the posterior probabilities (P(best))^10^, the best intervention is probably Venlafaxine and is very likely to be either Paroxetine. This agrees with the cumulative ranking probabilities (SUCRA values) under the ΝΜΑ^11^ (see also Figure 3). Placebo is the worst to deal with depression. However, ΝΜΑ RE meta-regression analysis according to the cumulative ranking probabilities suggests that Venlafaxine and Nefazodone are the best treatments in terms of efficacy (see Figure 4). In Figure 3 and Figure 4 the larger the area under the curve the larger the probability of the corresponding treatment is to be the best.

**Figure 3. Cumulative probabilities estimated under the ΝΜΑ in the Bayesian framework, when RMD is applied. The vertical axis represents the cumulative probability for each treatment to be the best option, whereas the horizontal axis represents the five possible ranks. The treatment with the largest area under its curve is most probably the best treatment.**

**Figure 4. Cumulative probabilities estimated under the ΝΜΑ RE meta-regression analysis in the Bayesian framework, when RMD is applied. The vertical axis represents the cumulative probability for each treatment to be the best option, whereas the horizontal axis represents the five possible ranks. The treatment with the largest area under its curve is most probably the best treatment.**

## Effect Size : Standardised Mean Difference (SMD)

|  | | **ΝΜΑ** | | | | |
| --- | --- | --- | --- | --- | --- | --- |
| **ΝΜΑ RE meta-regression analysis** | **(outlier excluded)** | **Placebo** | -0.22  (-0.41, -0.04) | -0.41  (-0.56,-0.25) | -0.27  (-0.41,-0.13) | -0.37  (-0.50,-0.24) |
|  |  | -0.26  (-0.43, -0.08) | **Fluoxetine** | -0.19  (-0.43,0.06) | -0.05  (-0.28, 0.19) | -0.15  (-0.37, 0.07) |
|  |  | -0.27  (-0.48, -0.05) |  |  |  |  |
|  |  | -0.41  (-0.56,-0.25) | -0.15  (-0.39,0.08) | **Venlafaxine** | 0.14  (-0.07, 0.35) | 0.03  (-0.17, 0.24) |
|  |  | -0.41  (-0.58,-0.25) | -0.15  (-0.42,0.12) |  |  |  |
|  |  | -0.29  (-0.43,-0.15) | -0.03  (-0.26, 0.19) | 0.12  (-0.09, 0.32) | **Nefazodone** | -0.10  (-0.30, 0.08) |
|  |  | -0.30  (-0.45,-0.15) | -0.03  (-0.31, 0.24) | 0.12  (-0.10, 0.33) |  |  |
|  |  | -0.35  (-0.48,-0.23) | -0.10  (-0.32, 0.12) | 0.05  (-0.14, 0.25) | -0.06  (-0.25, 0.13) | **Paroxetine** |
|  |  | -0.36  (-0.49,-0.23) | -0.10  (-0.34, 0.15) | 0.05  (-0.15, 0.26) | -0.07  (-0.27, 0.14) |  |

Table 11. Efficacy of all antidepressant drugs according to ΝΜΑ (light blue cells) and ΝΜΑ RE meta-regression analysis (blue cells). In dark blue cells the ΝΜΑ RE meta-regression analysis’ results are reported after the exclusion of the outlying study. Drugs are reported in order according to efficacy ranking. Comparisons between treatments should be read from left to right, where a negative value ($\boldsymbol{SMD<0}$) favours the column-defining treatment. Significant results are in bold.

Table 12. Order of treatments according to the P(best)^10^ under the frequentist approach and SUCRA Values along with their Credible Intervals (CrI) under the Bayesian approach^11^. P(Best) was computed from 10000 parametric bootstrap samples.

|  | Frequentist Approach  (P(best)) | Bayesian Approach  (SUCRA Values) | |
| --- | --- | --- | --- |
| Treatment | ΝΜΑ | ΝΜΑ  (CrI) | ΝΜΑ RE meta-regression analysis  (CrI) |
| Placebo | 0.00 | 0.00 (0.00, 0.00) | 0.00 (0.00, 0.00) |
| Fluoxetine | 0.05 | 0.37 (0.25, 1.00) | 0.41 (0.25, 1.00) |
| Venlafaxine | 0.40 | 0.87 (0.50, 1.00) | 0.87 (0.25, 1.00) |
| Nefazodone | 0.08 | 0.47 (0.25, 1.00) | 0.50 (0.25, 1.00) |
| Paroxetine | 0.54 | 0.79 (0.25, 1.00) | 0.71 (0.25, 1.00) |

According to the posterior probabilities (P(best))^10^ when SMD is applied, the two best interventions are probably Venlafaxine and Paroxetine. This agrees with the cumulative ranking probabilities (SUCRA values) under the ΝΜΑ^11^ (see Figure 5) and ΝΜΑ RE meta-regression analysis (see Figure 6). In Figure 5 and Figure 6 the larger the area under the curve the larger the probability of the corresponding treatment is to be the best.

Figure 5. Cumulative probabilities estimated under the ΝΜΑ in the Bayesian framework, when SMD is applied. The vertical axis represents the cumulative probability for each treatment to be the best option, whereas the horizontal axis represents the five possible ranks. The treatment with the largest area under its curve is most probably the best treatment.

Figure 6. Cumulative probabilities estimated under the ΝΜΑ RE meta-regression analysis in the Bayesian framework, when SMD is applied. The vertical axis represents the cumulative probability for each treatment to be the best option, whereas the horizontal axis represents the five possible ranks. The treatment with the largest area under its curve is most probably the best treatment.

# What is the role of the initial severity?

Table 13. Initial severity along with its 95% Confidence Interval under the frequentist approach and its 95% Credible Intervals (*CrI*) under the Bayesian approach. The heterogeneity is estimated under the DerSimonian and Laird (DL) in the Frequentist approach, whereas it is sampled from $\boldsymbol{\tau}^{\mathbf{2}}\boldsymbol{\sim N(0,1)I(0,)}$ in the Bayesian approach.

|  |  | Initial Severity ($\beta$) | |
| --- | --- | --- | --- |
|  |  | Raw Mean Difference | Standardised Mean Difference |
| Frequentist approach | Simple RE meta-regression analysis (DL) | 0.42 (0.31, 0.74) | 0.05 (0.01, 0.08) |
|  | Simple RE meta-regression analysis (DL)  (outlier excluded) | 0.53 (0.09, 0.97) | 0.05 (0.01, 0.08) |
| Bayesian approach | Simple RE meta-regression analysis | 0.41 (0.20, 0.62) | 0.03 (-0.01, 0.07) |
|  | Simple RE meta-regression analysis  (outlier excluded) | 0.41 (0.08, 0.75) | 0.03 (-0.01, 0.07) |
|  | ΝΜΑ RE meta-regression analysis | 0.38 (0.16, 0.61) | 0.03 (-0.01, 0.06) |
|  | ΝΜΑ RE meta-regression analysis (outlier excluded) | 0.46 (0.14, 0.78) | 0.03 (-0.02, 0.08) |

We observe that as the initial severity increases the effectiveness of interventions increases. The initial severity appears to be a statistically significant factor of the effectiveness in all methods under the RMD. However, when SMD is considered the initial severity appears to have marginally a non-statistically significant influence on the effectiveness of all treatments under the Bayesian methods and statistically significant influence under the frequentist methods.

The initial severity forms part of the definition of the SMD and there is a strong relationship between them. Thus, it is very likely that the significance under the frequentist approach is due to this relationship. The SMD is computed as the difference between the observed change-scores divided by the pooled standard deviation. However, the change score is computed as the difference between the initial severity and final values. Thus, even if there is no true relationship between initial severity and treatment efficacy, it is likely to be observed one.

On the other hand, the non-significance in Bayesian methods is accounted for the regression to the mean of the true change-score values. The contribution of the observational evidence and the potential biases are down-weighted. Bayesian methods allow us to examine the true relationship between the initial severity and treatment effectiveness.

Table 14. .Initial severity along with its 95% Credible Interval when the Raw Mean Difference and the Standardised Mean Difference are applied. Twelve different priors for $\boldsymbol{\tau}^{\mathbf{2}}$ are considered.

|  | Raw Mean Difference | | Standardised Mean Difference | |
| --- | --- | --- | --- | --- |
| Prior Distribution for heterogeneity | Simple RE meta-regression | ΝΜΑ RE meta-regression | Simple RE meta-regression | ΝΜΑ RE meta-regression |
| $1/{\tau^{2}}\sim Pareto(1,0.001)$ | 0.41 (0.16,0.66) | 0.39 (0.12, 0.66) | 0.03 (0.00,0.07) | 0.03 (-0.01, 0.06) |
| $1/{\tau^{2}}\sim Pareto(1,0.25)$ | 0.41 (0.17,0.66) | 0.39 (0.12, 0.65) | 0.03 (0.00, 0.07) | 0.03 (-0.01, 0.06) |
| $1/{\tau^{2}}\sim Gamma(0.01,0.01)$ | 0.41 (0.19,0.62) | 0.38 (0.16, 0.61) | 0.03 (0.00, 0.06) | 0.03 (-0.01, 0.06) |
| $1/{\tau^{2}}\sim Gamma(0.1,0.1)$ | 0.41 (0.20,0.62) | 0.38 (0.15, 0.62) | 0.04 (0.00, 0.07) | 0.03 (-0.01, 0.07) |
| $\tau\sim Uniform(0,100)$ | 0.41 (0.19,0.64) | 0.38 (0.14, 0.63) | 0.03 (0.00,0.06) | 0.03 (0.00, 0.06) |
| $\tau\sim Uniform(0,2)$ | 0.41 (0.18,0.64) | 0.38 (0.14, 0.63) | 0.03 (0.00,0.06) | 0.03 (-0.01, 0.06) |
| $\tau\sim N(0,100)$, $\tau>0$ | 0.41 (0.18,0.64) | 0.38 (0.14, 0.63) | 0.03 (0.00,0.06) | 0.03 (-0.01, 0.06) |
| $\tau\sim N(0,1)$, $\tau>0$ | 0.41 (0.20,0.62) | 0.38 (0.16, 0.61) | 0.03 (0.00,0.06) | 0.03 (-0.01, 0.06) |
| $\tau^{2}\sim Uniform(0,1000)$ | 0.41 (0.17,0.66) | 0.36 (0.11, 0.66) | 0.03 (0.00,0.06) | 0.03 (-0.01, 0.06) |
| $\tau^{2}\sim Uniform(0,4)$ | 0.41 (0.17,0.66) | 0.39 (0.12, 0.65) | 0.03 (0.00,0.07) | 0.03 (-0.01, 0.06) |
| $log(\tau^{2})\sim Uniform(-10,10)$ | 0.40 (0.21,0.60) | 0.39 (0.18, 0.59) | 0.03 (0.00,0.05) | 0.03 (0.00, 0.06) |
| $log(\tau^{2})\sim Uniform(-10,1.386)$ | 0.40 (0.21,0.60) | 0.38 (0.18, 0.59) | 0.03 (0.00,0.06) | 0.03 (0.00, 0.05) |

The Bayesian methods when the 12 different prior distributions placed on the heterogeneity and the RMD is the effect size suggest that the initial severity is statistically significant. However, when SMD is the effect size the initial severity is marginally non-significant.

| 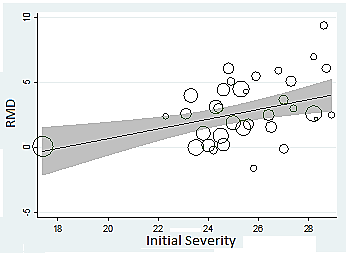 | 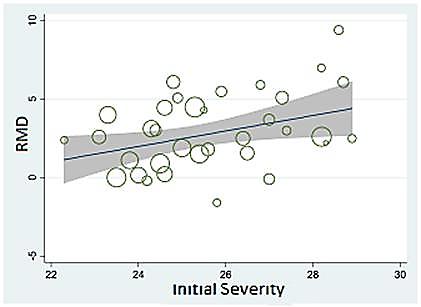 |
| --- | --- |
| Figure 7 RMD in 35 trials of all treatments, according to the Initial Severity. The area of each circle is proportional to the weight of the RMD estimate. $\left( \boldsymbol{y= -7.94+0.42\cdot x} \right)$ | Figure 8. RMD in 34 trials of all treatments, according to the Initial Severity. The area of each circle is proportional to the weight of the RMD estimate. $\left( \boldsymbol{y= -10.70+0.53\cdot x} \right)$ |
| 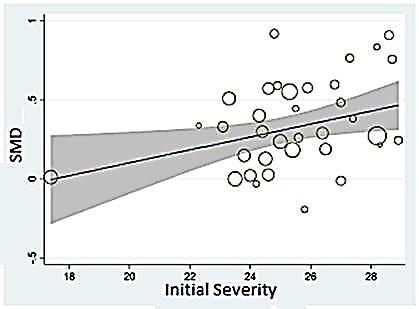 | 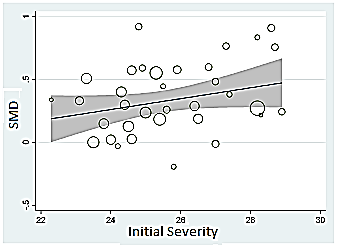 |
| Figure 9. SMD in 35 trials of all treatments, according to the Initial Severity. The area of each circle is proportional to the weight of the SMD estimate. $\left( \boldsymbol{y= -0.82+0.05\cdot x} \right)$ | Figure 10. SMD in 34 trials of all treatments, according to the Initial Severity. The area of each circle is proportional to the weight of the SMD estimate. $\left( \boldsymbol{y= -0.82+0.05\cdot x} \right)$ |

The examination of any association of the initial severity with the treatment effect, suggests that as the initial severity increases the effectiveness of the interventions increases (see Figure 7, Figure 9). The initial severity appears to have a statistically significant influence on the effectiveness of all treatments under both RMD and SMD in the Frequentist methods. Excluding the outlying trial we conclude to the same findings as earlier (see Figure 8, Figure 10).

# Is there a change in the difference between the active drug and placebo in more recent RCTs in comparison to older ones?

Table 15. Simple RE meta-regression analysis with two covariates (initial severity and publication year) under the frequentist approach

| Covariates | RMD | p-value under RMD | Heterogeneity under RMD | SMD | p-value under SMD | Heterogeneity under SMD |
| --- | --- | --- | --- | --- | --- | --- |
| Initial severity | 0.43  (0.10, 0.76) | 0.01 | 1.61 | 0.05  (0.00, 0.09) | 0.03 | 0.02 |
| Publication Year | 0.09  (-0.19,0.36) | 0.51 |  | 0.01  (-0.02,0.04) | 0.44 |  |

The heterogeneity has been reduced from 2.50 (Simple RE meta-analysis) to 1.61 (Simple RE meta-regression) under the RMD effect measure. While there is strong evidence for an association between initial severity and the effect of antidepressants (see also section 5), there is no evidence for an association with the year of publication.

The same picture is observed under the SMD effect measure, where the heterogeneity is reduced from 0.03 (Simple RE meta-analysis) to 0.02 (Simple RE meta-regression). While there is evidence for an association between initial severity and the effect of antidepressants, there is no evidence for an association with the year of publication.

# References

(1) Sutton, A. J.; Abrams, K. R. Bayesian Methods in Meta-Analysis and Evidence Synthesis. *Stat Methods Med. Res.* **2001,** *10,* 277-303.

(2) Lu, G.; Ades, A. E. Combination of Direct and Indirect Evidence in Mixed Treatment Comparisons. *Stat. Med.* **2004,** *23,* 3105-3124.

(3) Caldwell, D. M.; Ades, A. E.; Higgins, J. P. Simultaneous Comparison of Multiple Treatments: Combining Direct and Indirect Evidence. *BMJ* **2005,** *331,* 897-900.

(4) Higgins, J. P.; Whitehead, A. Borrowing Strength From External Trials in a Meta-Analysis. *Stat. Med.* **1996,** *15,* 2733-2749.

(5) Lambert, P. C.; Sutton, A. J.; Burton, P. R.; Abrams, K. R.; Jones, D. R. How Vague Is Vague? A Simulation Study of the Impact of the Use of Vague Prior Distributions in MCMC Using WinBUGS. *Stat Med.* **2005,** *24,* 2401-2428.

(6) Friedrich, J. O.; Adhikari, N. K.; Beyene, J. Ratio of Means for Analyzing Continuous Outcomes in Meta-Analysis Performed As Well As Mean Difference Methods. *J. Clin. Epidemiol.* **2011,** *64,* 556-564.

(7) Song, F.; Sheldon, T. A.; Sutton, A. J.; Abrams, K. R.; Jones, D. R. Methods for Exploring Heterogeneity in Meta-Analysis. *Eval. Health Prof.* **2001,** *24,* 126-151.

(8) Deeks, J. J. Issues in the Selection of a Summary Statistic for Meta-Analysis of Clinical Trials With Binary Outcomes. *Stat. Med.* **2002,** *21,* 1575-1600.

(9) Engels, E. A.; Schmid, C. H.; Terrin, N.; Olkin, I.; Lau, J. Heterogeneity and Statistical Significance in Meta-Analysis: an Empirical Study of 125 Meta-Analyses. *Stat. Med.* **2000,** *19,* 1707-1728.

(10) White, I. R. Multivariate Random-Effects Meta-Regression: Updates to Mvmeta. *Stata Journal* **2011,** *11,* 255-270.

(11) Salanti, G.; Ades, A. E.; Ioannidis, J. P. Graphical Methods and Numerical Summaries for Presenting Results From Multiple-Treatment Meta-Analysis: an Overview and Tutorial. *J Clin. Epidemiol.* **2011,** *64,* 163-171.
